# GEF-H1 controls focal adhesion signaling that regulates mesenchymal stem cell lineage commitment

**DOI:** 10.1242/jcs.150227

**Published:** 2014-10-01

**Authors:** I-Husan Huang, Cheng-Te Hsiao, Jui-Chung Wu, Rong-Fong Shen, Ching-Yi Liu, Yang-Kao Wang, Yu-Chen Chen, Chi-Ming Huang, Juan C. del álamo, Zee-Fen Chang, Ming-Jer Tang, Kay-Hooi Khoo, Jean-Cheng Kuo

**Affiliations:** 1 Institute of Biochemistry and Molecular Biology, National Yang-Ming University, Taipei 11221, Taiwan; 2 Institute of Biochemical Sciences, National Taiwan University, Taipei 10617, Taiwan; 3 Institute of Biological Chemistry, Academia Sinica, Taipei 11529, Taiwan; 4 Proteomics and Analytical Biochemistry Unit, Research Resources Branch, National Institute on Aging, NIH, Baltimore, MD 21224, USA; 5 Institute of Basic Medical Sciences, National Cheng Kung University, Tainan 70101, Taiwan; 6 Department of Physiology, National Cheng Kung University, Tainan 70101, Taiwan; 7 Department of Cell Biology and Anatomy, National Cheng Kung University, Tainan 70101, Taiwan; 8 Center for Neurotrauma and Neuroregeneration, Taipei Medical University, Taipei 11031, Taiwan; 9 Institute of Engineering in Medicine, University of California at San Diego, La Jolla, CA 92093, USA; 10 Department of Mechanical and Aerospace Engineering, University of California at San Diego, La Jolla, CA 92093, USA

**Keywords:** Focal adhesions, Mesenchymal stem cell, Osteogenesis, Stress fiber

## Abstract

Focal adhesions (FAs) undergo maturation that culminates in size and composition changes that modulate adhesion, cytoskeleton remodeling and differentiation. Although it is well recognized that stimuli for osteogenesis of mesenchymal stem cells (MSCs) drive FA maturation, actin organization and stress fiber polarization, the extent to which FA-mediated signals regulated by the FA protein composition specifies MSC commitment remains largely unknown. Here, we demonstrate that, upon dexamethasone (osteogenic induction) treatment, guanine nucleotide exchange factor H1 (GEF-H1, also known as Rho guanine nucleotide exchange factor 2, encoded by *ARHGEF2*) is significantly enriched in FAs. Perturbation of GEF-H1 inhibits FA formation, anisotropic stress fiber orientation and MSC osteogenesis in an actomyosin-contractility-independent manner. To determine the role of GEF-H1 in MSC osteogenesis, we explore the GEF-H1-modulated FA proteome that reveals non-muscle myosin-II heavy chain-B (NMIIB, also known as myosin-10, encoded by *MYH10*) as a target of GEF-H1 in FAs. Inhibition of targeting NMIIB into FAs suppresses FA formation, stress fiber polarization, cell stiffness and osteogenic commitments in MSCs. Our data demonstrate a role for FA signaling in specifying MSC commitment.

## INTRODUCTION

Mesenchymal stem cells (MSCs) derived from bone marrow are multi-potent cells that serve as an attractive cell source for cell therapy in the treatment of diseases or injury (Brooke et al., 2007; Uccelli et al., 2008). MSCs have the potential to differentiate into a variety of cell types, including osteoblasts, chrondrocytes, adipocytes, myoblasts and nerves (Deng et al., 2006; Hofstetter et al., 2002; Horwitz et al., 2005; Kondo et al., 2005; McBeath et al., 2004; Pittenger et al., 1999; Uccelli et al., 2008). Commitment of MSCs to the osteoblast fate is known to be induced by soluble factors, for example, bone morphogenetic proteins and dexamethasone (Dex) (Chen et al., 2011; Mikami et al., 2011; Oshina et al., 2007; Wang et al., 2012), or by tension from bone-level matrix elasticity (Engler et al., 2006; Swift et al., 2013). These stimuli can activate RhoA- and Rho-associated protein kinase (ROCK)-mediated signaling pathways, which increase myosin light chain (MLC) phosphorylation at Thr18/Ser19, thereby generating myosin-II-mediated contractile force, and subsequent remodeling of actin filaments and maturation of focal adhesions (FAs) (Chen et al., 2011; Wang et al., 2012). Thus, during osteogenic differentiation, MSCs change their cell morphology from a fibroblast-like phenotype to a near spherical shape, remodel actin cytoskeleton networks, promote stress fiber formation, and mature FAs. Importantly, the spread cell shape, cytoskeletal organization and mature FAs are crucial in supporting osteogenic differentiation of MSCs (Engler et al., 2006; Müller et al., 2013; Rodríguez et al., 2004), whereas round, un-spread MSCs undergo adipogenic differentiation (McBeath et al., 2004; Swift et al., 2013). Therefore, cell shape and cytoskeletal mechanics mediate the commitment of MSCs to the osteoblast or adipocyte lineages.

FAs are integrin-based adhesive organelles at cell membrane that are necessary for cells to adhere, sense and transduce biochemical or physical signals (Burridge et al., 1988; Hynes, 2002; Jockusch et al., 1995; Schwartz et al., 1995). FAs start to form when their central component, integrin receptor, is activated by engagement with the extracellular matrix (ECM) and then recruits FA-associated proteins to connect with the actin cytoskeleton (Burridge et al., 1988; Hynes, 2002; Jockusch et al., 1995; Schwartz et al., 1995; Zaidel-Bar and Geiger, 2010; Zaidel-Bar et al., 2007). Evidence indicates that the size and composition of FAs are regulated spatiotemporally in a process called FA maturation (Chrzanowska-Wodnicka and Burridge, 1996; Kuo, 2013; Kuo et al., 2011; Pletjushkina et al., 1998; Riveline et al., 2001). During maturation, FAs grow in size and change composition, after which they either stabilize or begin to disassemble. Modulation of the maturation state of FAs plays a determinant role in specifying MSC differentiation, given that different maturation states of FAs modulated by tissue-level ECM elasticity lead MSCs differentiation into different cell types, such as neuronal, muscle or bone cells (Engler et al., 2006). A proteomic study has elucidated the hierarchical cascade of FA compositional changes during FA maturation, indicating that the maturation process modulates the abundance of FA-associated proteins that transduce distinct biological signals (Kuo et al., 2011). The components in mature FAs mediate FA strengthening and the formation of actin bundles (stress fibers) (Kuo, 2013; Kuo et al., 2011), suggesting that FA proteins control the remodeling of actin cytoskeleton networks and further control commitment of MSCs.

Guanine nucleotide exchange factor H1 (GEF-H1, also known as Rho guanine nucleotide exchange factor 2, encoded by *ARHGEF2*) has been identified as a GEF protein with activity towards RhoA (Ren et al., 1998), which is known to promote myosin-II-driven contractile force and stress fiber formation (Bishop and Hall, 2000; Sahai and Marshall, 2002). The activity of GEF-H1 is known to be regulated by microtubule dynamics (Krendel et al., 2002). GEF-H1 activity is inhibited by association with polymerized microtubules and is switched on upon microtubule disassembly (Krendel et al., 2002). In the proteomic analysis, GEF-H1 had been identified as concentrating in mature FAs (Kuo et al., 2011), implying that GEF-H1 might serve as a molecular link between mature FAs and actin cytoskeleton organization.

Here, we examined whether GEF-H1 can modulate the FA composition that controls commitment of MSCs to osteoblast lineages, and if so, how. We used MSCs as model system, because it has been documented that the cells can not only differentiate into osteoblasts or adipocytes (Caplan, 1991; Friedenstein, 1976; Pittenger et al., 1999), but also show FA maturation, actin cytoskeleton organization and stress fiber polarization during osteogentic differentiation (Chen et al., 2011; Fu et al., 2010; Wang et al., 2012). The commercialized osteogenesis induction medium (OIM) contains 0.1 µM Dex, whereas there is 1 µM Dex in adipogenesis induction medium (AIM) (Mikami et al., 2011; Oshina et al., 2007). In response to 0.1 µM Dex, we found that GEF-H1 was enriched in mature FAs of MSCs. By examining the effects of GEF-H1 on FA composition, we have identified that GEF-H1 serves as a scaffold to mediate the recruitment of non-muscle myosin-II heavy chain-B (NMIIB, encoded by *MYH10*) to FAs, which is an important step required for stress fiber polarization, FA formation and control of the commitment of MSCs to the osteogenic lineage.

## RESULTS

### Microtubule stabilization influences Dex-regulated cytoskeletal architecture

To test the notion that stimuli for osteogenic differentiation of MSCs regulate the organization of actin cytoskeleton and FAs in MSCs, we first examined the effects of Dex, the main component in the OIM for osteogenic differentiation, on the formation of stress fibers and FAs in MSCs. Immunolocalization of F-actin and the FA marker paxillin showed that Dex treatment (0.1 µM, 6 h) significantly induced stress fiber formation and increased FA number (supplementary material Fig. S1A–C). Thus, MSCs responded to Dex through reorganization of the cytoskeleton to produce intensive stress fibers and mature FAs.

We next examined the effects of microtubule dynamics on Dex-induced actin cytoskeleton organization. Microtubule dynamics were stabilized with taxol treatment. Immunolocalization of F-actin and paxillin revealed that taxol treatment led to remodeling of the orientation of stress fibers and mature FAs, but did not completely abolish their formation (supplementary material Fig. S1D). GEF-H1 has been shown to link microtubule and actin cytoskeleton dynamics (Krendel et al., 2002), which would imply that Dex-induced stress fiber polarization might be GEF-H1 dependent.

### MSCs commitment depends on GEF-H1 expression

**Fig. 1.**
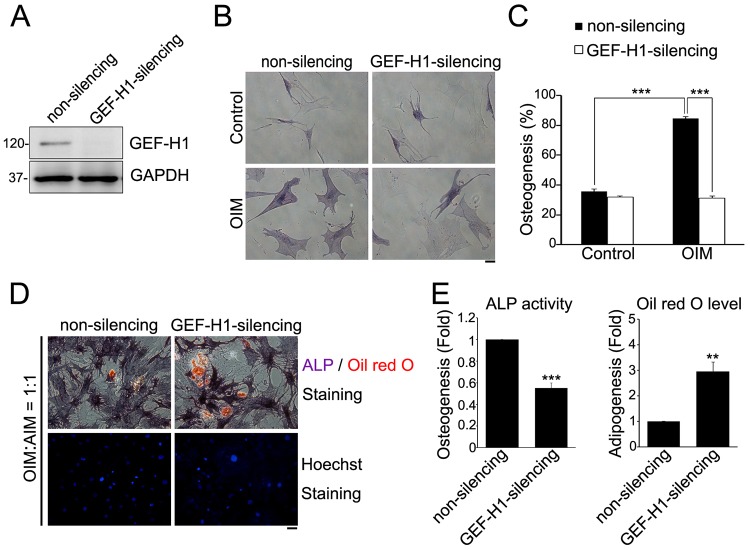
**MSCs osteogenesis versus adipogenesis depends on GEF-H1 expression.** (A) The expression of GEF-H1 and GAPDH in non-silencing and GEF-H1-silencing MSCs was analyzed by western blotting. (B) Non-silencing and GEF-H1-silencing MSCs were treated with control culture medium or osteogenesis induction medium (OIM) for 14 days and stained for the activity of ALP. Scale bar: 50 µm. (C) The percentage of cells showing osteogenesis (ALP-positive cells). Data are mean±s.e.m. [non-silencing MSCs: *n* = 60 cells (control); *n* = 111 cells (OIM); GEF-H1-silencing MSCs: *n* = 82 cells (control); *n* = 77 cells (OIM)]. ***P<0.0001. (D) Non-silencing and GEF-H1-silencing MSCs were treated with mixed differentiation medium (OIM∶AIM = 1∶1) for 14 days, and stained for the presence of lipid (Oil Red O; red), the activity of ALP (purple) and Hoechst 33342 (blue). Scale bar: 50 µm. (E) Fold change in cells showing osteogenesis (ALP activity) or adipogenesis (Oil Red O level) in GEF-H1-silencing MSCs relative to non-silencing MSCs. Data are mean±s.e.m (non-silencing: *n* = 571 cells; GEF-H1-silencing: *n* = 348 cells). ***P<0.0001, **P<0.005.

Evidence indicates that the spread cell shape, cytoskeletal organization and mature FAs support MSC osteogenic differentiation (Engler et al., 2006; McBeath et al., 2004; Müller et al., 2013; Rodríguez et al., 2004). To confirm the effect of GEF-H1 in MSC osteogenic differentiation, we generated non-silencing and GEF-H1-silencing MSCs using lentiviral short hairpin RNA (shRNA) (Fig. 1A). The cells were cultured in OIM for 14 days and stained for alkaline phosphatase (ALP) activity, a marker of osteogenesis. In OIM-stimulated MSCs, non-silencing MSCs expressed more ALP than GEF-H1-silencing MSCs (Fig. 1B). We quantified the degree of osteogenesis by comparing the percentages of cells stained with ALP, and found that OIM promoted ALP labeling of the non-silencing MSCs by ∼50% but had no significant effect on that of GEF-H1-silencing MSCs, indicating the importance of GEF-H1 expression on MSC osteogenesis (Fig. 1C). To further determine the effect of GEF-H1 on MSC differentiation to the osteogenic or adipogenic lineage, the non-silencing and GEF-H1-silencing MSCs were cultured in a mixed differentiation medium (OIM∶AIM = 1∶1) for 14 days (McBeath et al., 2004), and stained for ALP activity or lipid droplets, markers of osteogenesis and adipogenesis, respectively. In mixed differentiation medium, more non-silencing MSCs showed ALP activity, whereas more GEF-H1-silencing MSCs contained lipid droplets (Fig. 1D). We quantified the degree of osteogenesis and adipogenesis, and showed that silencing of GEF-H1 inhibited osteogenic commitment, but induced adipogenesis (Fig. 1E), suggesting that GEF-H1 expression promotes MSC osteogenic differentiation.

### GEF-H1 regulates actin cytoskeletal architecture

**Fig. 2.**
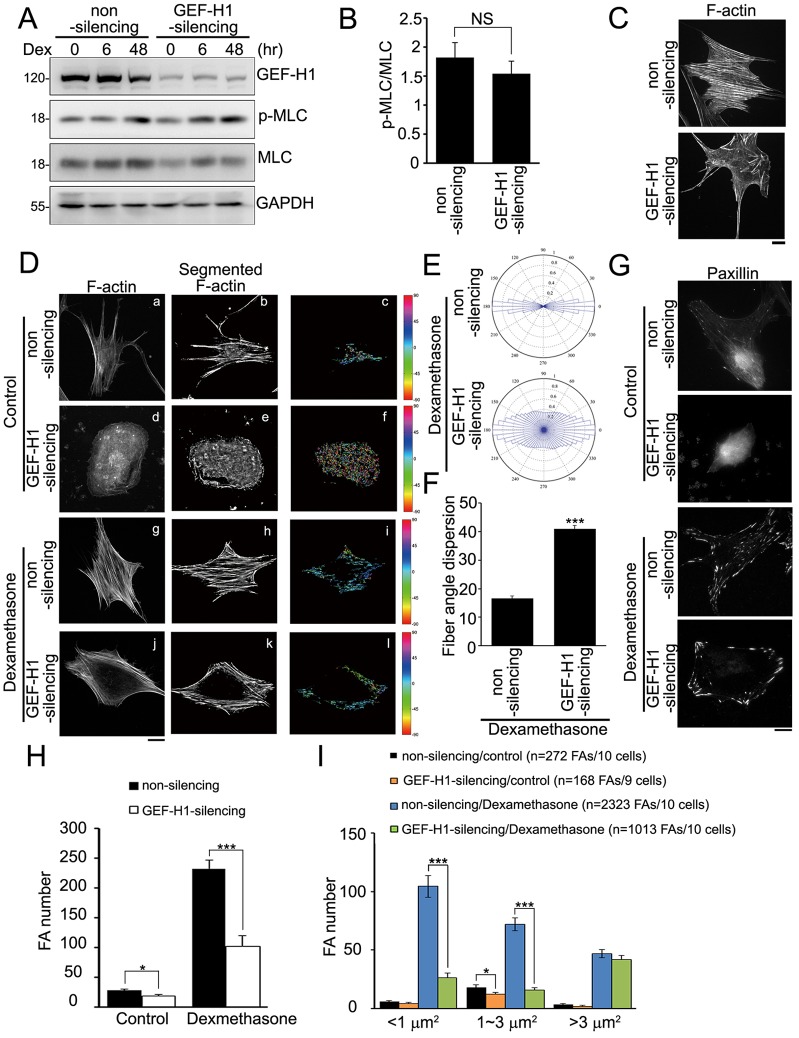
**GEF-H1 is not required for Dex-induced myosin II activation but mediates the organization of stress fibers orientation and FA formation.** (A) Cell lysate from serum-starved non-silencing and GEF-H1-silencing MSC-3A6 cells treated with Dex (0.1 µM) for 0, 6 or 48 h were analyzed by western blotting. p-MLC, phosphorylated MLC. (B) Densitometry analysis of western blots showing the relative levels of the ratio of phosphorylated MLC to total MLC for non-silencing and GEF-H1-silencing cells treated with Dex (0.1 µM) for 6 h. Data are mean±s.e.m. (*n* = 3 for each condition). NS, not significant. (C) Serum-starved non-silencing and GEF-H1-silencing MSCs treated with Dex (0.1 µM) and nocodazole (10 µM) for 6 h were immunostained with FITC–phalloidin. Scale bar: 20 µm. (D) Serum-starved non-silencing and GEF-H1-silencing MSCs treated with ethanol (control) or Dex (0.1 µM) for 6 h were immunostained with FITC–phalloidin, to localize F-actin (a, d, g and j). Scale bar: 20 µm. After image processing to show the segmented F-actin, the image was rotated to place the median stress-fiber-orientation as horizontal (b, e, h and k). The orientation of each fiber was depicted with a specific color as indicated by the color bar at the right-hand side of each panel (c, f, i and l). (E) The polar histograms and (F) spread distribution of stress fiber orientations were statistically calculated from non-silencing (*n* = 15 cells) and GEF-H1-silencing MSCs (*n* = 15 cells) treated with Dex. Data are mean±s.e.m. ***P<0.0001. (G) Serum-starved non-silencing and GEF-H1-silencing MSCs treated with ethanol (control) or Dex (0.1 µM) for 6 h were immunostained with paxillin, to visualize FAs, and imaged by TIRF microscopy. Scale bar: 20 µm. (H) The number of segmented paxillin-marked FAs of MSCs, as described in G. Data are mean±s.e.m. (*n* = 10 cells for each condition). *P<0.05; ***P<0.0001. (I) Size distribution of segmented paxillin-marked FAs of MSCs, as described in G. Data are mean±s.e.m. *P<0.05, ***P<0.0001.

GEF-H1 has been shown to promote stress fiber formation and FA maturation through RhoA and ROCK signaling pathways, resulting in increased levels of MLC phosphorylation at Thr18/Ser19 and myosin II activity (Chang et al., 2008; Chrzanowska-Wodnicka and Burridge, 1996; Krendel et al., 2002; Ridley and Hall, 1992). We next examined whether GEF-H1 was involved in Dex-induced myosin II activation (phosphorylation at Thr18/Ser19 of MLC) in MSC-3A6 cells. Non-silencing and GEF-H1-silencing cells were treated with 0.1 µM Dex for 0, 6 or 48 h, and cellular MLC phosphorylation was analyzed by western blotting. We found that the levels of MLC phosphorylation were increased after 48 h Dex treatment in both non-silencing and GEF-H1-silencing cells, compared with 0 h Dex treatment (Fig. 2A). Quantitatively, silencing of GEF-H1 did not suppress the levels of MLC phosphorylation after 6 h Dex treatment (Fig. 2B). To analyze the ability of GEF-H1 in the organization of the actin cytoskeleton, Dex-stimulated non-silencing and GEF-H1-silencing MSCs were treated with the microtubule-depolymerizing drug nocodazole to observe the pattern of F-actin. The results revealed that the depletion of GEF-H1 substantially decreased actin stress fiber formation (Fig. 2C). However, without the treatment of nocodazole, a slight suppression of Dex-induced stress fiber formation in GEF-H1-silencing MSCs was shown, in comparison with that in non-silencing cells (Fig. 2D), suggesting that GEF-H1 modulates MSCs lineage commitment through a mechanism that is yet to be determined.

Surprisingly, we found that, upon Dex treatment, silencing of GEF-H1 expression altered the orientation of the stress fibers (Fig. 2Dg and Fig. 2Dj) in a similar manner to that observed upon taxol treatment (supplementary material Fig. S1D), whereas only a few stress fibers were observed in non-silencing and GEF-H1-silencing MSCs without Dex stimulation (Fig. 2Da and Fig. 2Dd). To calculate the orientation of stress fibers, we analyzed the images of F-actin using in-house MATLAB scripts (Karlon et al., 1999). To compare the distributions of orientation, the cell image with the segmented stress fibers was rotated to set the median stress fiber orientation to horizontal axis (0°) (Fig. 2Db, e, h and k). The color map displayed the orientation of the rest of stress fibers with respect to the median stress fiber orientation, shown as an angle between −90° and 90° (Fig. 2Dc, f, i and l). We further calculated the spread of the distribution of stress fiber orientation upon Dex treatment to provide a measure of the degree of stress fiber polarization with the standard deviation (Fig. 2E) and the level of fiber angle dispersion (Fig. 2F). Non-silencing MSCs possessed higher degrees of aligned stress fiber orientations, whereas GEF-H1-silencing MSCs displayed wider distribution of stress fiber orientations (Fig. 2E,F). These results suggest that GEF-H1 expression regulates the anisotropic orientation of stress fibers in response to Dex.

We next determined the role of GEF-H1 in Dex-induced FA formation (supplementary material Fig. S1A–C; Fig. 2G,H). Immunolocalization of paxillin in Dex-treated MSCs showed that silencing of GEF-H1 significantly decreased the FA number in MSCs, in comparison with that in the non-silencing MSCs (Fig. 2G,H). Quantitative analysis of paxillin-marked FAs indicated that silencing of GEF-H1 decreased the number of medium-sized FAs (1∼3 µm^2^) and small FAs (<1 µm^2^), but had no effect on large FAs (>3 µm^2^) (Fig. 2I). Thus, GEF-H1 appears to mediate Dex-induced FA formation.

### Dex increases the recruitment of GEF-H1 into FAs

**Fig. 3.**
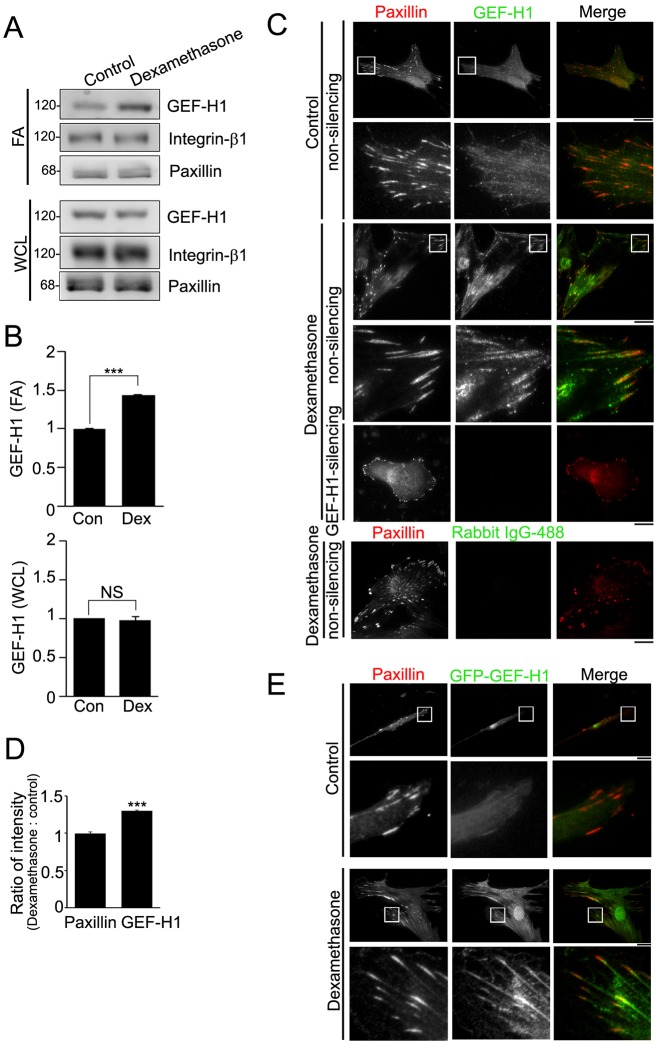
**GEF-H1 is enriched in Dex-stimulated FAs.** (A) FA fraction (FA) and whole-cell lysate (WCL) from serum-starved MSC-3A6 cells treated with ethanol (control) or Dex (0.1 µM) for 6 h were analyzed by western blotting. (B) Densitometry analysis of western blots showing the ratio of GEF-H1 in FA and WCL from MSC-3A6 cells treated with Dex relative to ethanol (Con). Data are mean±s.e.m. (*n* = 3 for each condition). ***P<0.0001. NS, not significant. (C) TIRF images of immunolocalized paxillin (red) and GEF-H1 (green) or anti-rabbit-IgG antibody conjugated to Alexa Fluor 488 (green) in serum-starved non-silencing or GEF-H1-silencing MSCs treated with ethanol (control) or Dex (0.1 µM) for 6 h. Scale bars: 20 µm. The boxed 20 µm×20 µm areas indicated in the upper image rows are magnified in the row below. (D) Ratio of average density (intensity per µm^2^) of paxillin or GEF-H1 within segmented FAs of non-silencing MSCs treated with Dex relative to control. Data are mean±s.e.m. (*n* = 10 cells for each condition). ***P<0.0001. (E) Serum-starved MSCs overexpressing GFP–GEF-H1 (green) and treated with ethanol (control) or Dex (0.1 µM) for 6 h were immunostained for paxillin (red). Scale bars: 20 µm. The boxed 20 µm×20 µm areas indicated in the upper image rows are magnified in the row below.

Previous proteomic analysis has indicated that there is elevated levels of GEF-H1 in mature FAs (Kuo et al., 2011), revealing that it might enrich in FAs of MSCs. We then analyzed the levels of GEF-H1 in whole-cell lysate and isolated FA fractions of MSC-3A6 cells treated with ethanol (control) or Dex (0.1 µM) for 6 h. The results showed that treatment with Dex had no effect on the level of GEF-H1 in whole-cell lysate, but resulted in an ∼50% increase of GEF-H1 level in FAs (Fig. 3A,B). Immunolocalization of GEF-H1 and paxillin indicated that GEF-H1 was localized at paxillin-marked FAs in Dex-treated MSCs (Fig. 3C). Quantifying the ratio of fluorescence density of paxillin and GEF-H1 in FAs showed that Dex stimulation did not affect paxillin FA density, but significantly (∼30%) increased the FA-localized density of GEF-H1 (Fig. 3D). Analysis of GFP–GEF-H1 revealed that Dex treatment caused the association of GFP–GEF-H1 with paxillin-marked FAs (Fig. 3E). Thus, GEF-H1 is enriched in FAs of MSCs stimulated with Dex.

### The development of GEF-H1-modulated FA proteome by proteomic analysis

To further characterize the effects of GEF-H1 in Dex-stimulated FAs, we analyzed the composition and abundance of proteins in FAs from non-silencing and GEF-H1-silencing MSC-3A6 cells (supplementary material Fig. S2A). Serum-starved non-silencing and GEF-H1-silencing MSC-3A6 cells were treated with Dex (0.1 µM, 6 h) and hypotonically shocked to isolate FAs using the FA isolation method (Kuo et al., 2011; Kuo et al., 2012), which has been previously demonstrated to preserve the native FA organization and size. The isolated FA fractions were subject to liquid chromatography (LC)-tandem mass spectrometry (MS/MS) analysis. The proteins that were reproducibly identified at least two out of five replicate runs (five independent experimental runs for each condition) were included into the reproducible lists, which contain 321 and 250 proteins in non-silencing (supplementary material Table S1) and GEF-H1-silencing FAs (supplementary material Table S2), respectively. In total, 335 proteins make the list under both conditions (85 proteins only in non-silencing FAs, 14 proteins only in GEF-H1-silencing FAs and 236 proteins in both conditions).

Based on the Gene Ontology (GO) and literature analysis, proteins in the reproducible lists were classified into six categories: focal adhesion, cytoskeleton, extracellular matrix, plasma membrane, cytoplasm, and uncharacterized. The category ‘focal adhesion’ only contained the proteins listed in the integrin adhesome (Zaidel-Bar and Geiger, 2010; Zaidel-Bar et al., 2007), which is the current list of most reported FA molecules from different forms of integrin-mediated FAs, although new FA components have been frequently identified. In supplementary material Fig. S2B, the pie diagrams summarize the percentage of proteins in these categories, and show that the percentage distributions of these proteins in all categories were similar between non-silencing or GEF-H1-silencing FA fractions. Although the reproducibly identified proteins might contain uncharacterized, undiscovered and transient FA proteins, or contaminants, our main focus was the determination of composition changes of FAs modulated by GEF-H1 expression.

To characterize the effects of GEF-H1 on the abundance changes of FA proteins, we evaluated the relative levels of individual FA protein isolated from non-silencing and GEF-H1-silencing MSC-3A6 cells using their spectrum counts. Owing to the experimental variations, the raw spectrum count of each protein was normalized before further calculation (supplementary material Fig. S2A), as described previously (Kuo et al., 2011; Kuo et al., 2012). Finally, the relative abundance of each protein in FAs was expressed as a ratio of protein abundance. The 335 reproducibly identified proteins were classified by the magnitude of their ratio to form the GEF-H1-modulated FA proteome (supplementary material Table S3). They included 117 proteins with a ratio >2, indicating GEF-H1-dependent FA recruitment; 93 proteins with a ratio <0.5, indicating FA recruitment inhibited by GEF-H1 expression; and 125 proteins with a ratio between 0.5 and 2, indicating GEF-H1-independent FA recruitment. Thus, GEF-H1 affects FA enrichment of proteins leading to substantial changes in FA composition that might modulate distinct pathways in Dex-induced MSCs FA formation and stress fiber polarization.

Actin cytoskeleton mechanics in a living cell are known to be regulated dynamically by positive and negative regulators. In the GEF-H1-modulated FA proteome (supplementary material Fig. S3A), we found the proteins that served as positive regulators of actin mechanics were enriched in FAs of non-silencing cells. These proteins included NMIIB (*MYH10*), myosin essential light chain (MELC; *MYL6*), myosin regulatory light chain (MRLC; *MYL9*), myosin phosphatase (MYPT; *PPP1R12A*) and tropomyosin II (also known as β-tropomyosin, encoded by *TPM2*). However, the negative regulator caldesmon 1 (*CALD1*) (Grosheva et al., 2006) was concentrated in GEF-H1-silencing FAs. Therefore, GEF-H1 might control actin mechanics through modulating FA accumulation of these regulators.

Notably, we identified several factors known in GEF-H1-related signaling pathways from the GEF-H1-modulated FA proteome. In supplementary material Fig. S3B, we list 41 known GEF-H1-interacting proteins, categorized by their cellular localizations, e.g. focal adhesion, cytoskeleton and cytoplasm. Of the 41 GEF-H1-interacting proteins, only Rac1 and RhoA are known FA components listed in the integrin adhesome (Zaidel-Bar and Geiger, 2010; Zaidel-Bar et al., 2007), yet we reproducibly identified six GEF-H1-interacting proteins in the isolated FA fractions, including Rac1 (*RAC1*), non-muscle myosin-II heavy chain-A (NMIIA; *MYH9*), NMIIB (*MYH10*), vasolin-containing protein (VPC), 14-3-3τ (*YWHAQ*) and 14-3-3ζ (*YWHAZ*). NMIIA and NMIIB are different isoforms of non-muscle myosin II (NMII), and we detected more spectrum counts of NMIIA than that of NMIIB in non-silencing FA fractions, suggesting that NMIIA is more abundant than NMIIB at FAs. Although both NMIIA and NMIIB were detected in the FA fractions of non-silencing MSCs (supplementary material Table S1), only NMIIA was detected in the FA fractions of GEF-H1-silencing MSCs (supplementary material Table S2), indicating that silencing of GEF-H1 decreased the abundance of NMIIB at FAs, but not that of NMIIA. Furthermore, NMIIB is known to be involved in the signaling pathway downstream of RhoA-mediated actomyosin contractility (supplementary material Fig. S3C) (Chrzanowska-Wodnicka and Burridge, 1996; Ridley and Hall, 1992). Therefore, we focused on NMIIB to further determine how GEF-H1 regulates Dex-induced FA formation and stress fiber polarization in MSCs through FA signaling.

### GEF-H1 mediates FA recruitment of NMIIB

**Fig. 4.**
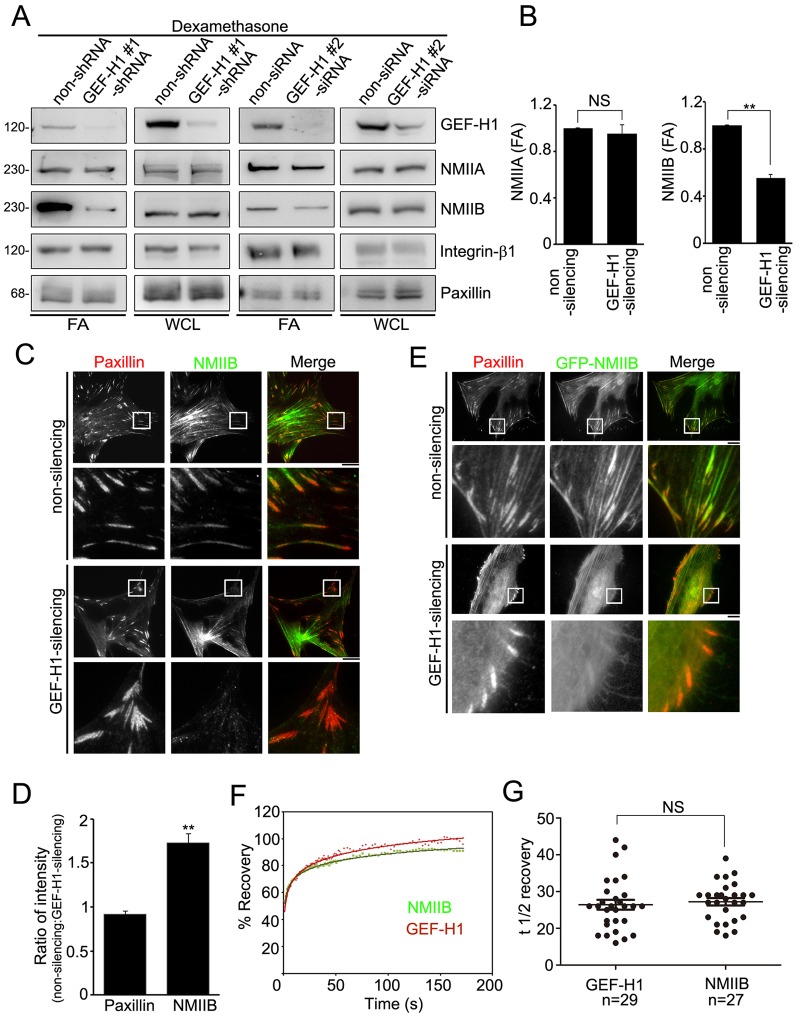
**GEF-H1 mediates the recruitment of NMIIB in FAs.** (A) FA fraction (FA) and whole-cell lysate (WCL) from serum-starved non-silencing (non-shRNA and non-siRNA) and GEF-H1-silencing (GEF-H1#1 shRNA and GEF-H1#2 siRNA indicate different sequence targets of GEF-H1) MSC-3A6 cells treated with Dex (0.1 µM, 6 h) were analyzed by western blotting. (B) Densitometry analysis of western blots showing the ratio of NMIIA or NMIIB in the FA fraction (FA) from MSC-3A6 cells expressing GEF-H1-silencing shRNA relative to non-silencing shRNA. Data are mean±s.e.m. (*n* = 3 for each condition). **P<0.005; NS, not significant. (C) TIRF images of immunolocalized paxillin (red) and NMIIB (green) in non-silencing and GEF-H1-silencing MSCs treated with Dex (0.1 µM, 6 h). Scale bar: 20 µm. The boxed 20 µm×20 µm areas indicated in the upper image rows are magnified in the row below. (D) The ratio of the average density (intensity per µm^2^) of paxillin or NMIIB within segmented FAs of MSCs expressing non-silencing shRNA relative to GEF-H1-silencing shRNA. Data are mean±s.e.m. (*n* = 8 cells for each condition). **P<0.005. (E) Serum-starved non-silencing and GEF-H1-silencing MSCs overexpressing GFP–NMIIB (green) and treated with Dex (0.1 µM) for 6 h were immunostained for paxillin (red). Scale bar: 20 µm. The boxed 20 µm×20 µm areas indicated in the upper image rows are magnified in the row below. (F,G) GFP–GEF-H1 and GFP–NMIIB localized to FAs were subjected to FRAP. Sample fluorescence recovery curves for GEF-H1 and NMIIB in a single FA (F). Half-times of fluorescence recovery (G). Data are mean±s.e.m. (*n* = number of FAs). NS, not significant.

To understand how GEF-H1-mediated FA signaling regulated stress fiber polarization, we focused on NMIIB. Immunoblotting of FA fractions from non-silencing and GEF-H1-silencing MSC-3A6 cells confirmed the positive regulation of NMIIB accumulation by GEF-H1 expression, whereas total NMIIB were not changed in GEF-H1-silencing MSCs (Fig. 4A), indicating that NMIIA was the major isoform in FAs of GEF-H1-silencing MSCs. In Fig. 4B, silencing of GEF-H1 resulted in a 40% decrease of NMIIB in FAs, with negligible effect on NMIIA, consistent with results seen in the GEF-H1-modulated FA proteome (supplementary material Table S3). Total internal reflection fluorescence (TIRF) microscopy analysis revealed an accumulation of NMIIB in the FAs of non-silencing MSCs but not in those of GEF-H1-silencing MSCs (Fig. 4C). Quantification of the ratio of fluorescence densities of paxillin and NMIIB in FAs indicated that GEF-H1 expression did not change the density of paxillin at FAs, but significantly increased the FA density of NMIIB (70%) (Fig. 4D). Analysis of GFP–NMIIB in Dex-treated non-silencing and GEF-H1-silencing MSCs confirmed that NMIIB concentration in paxillin-marked FAs appeared to be positively regulated by GEF-H1 (Fig. 4E). We next hypothesized that GEF-H1 and NMIIB had similar protein turnover within FAs upon Dex treatment. To test this, we used fluorescence recovery after photobleaching (FRAP) of GFP–GEF-H1 or GFP–NMIIB in single FAs (Fig. 4F) and calculated the mean fluorescence recovery *t*_1/2_ as the stability of FA binding (Bulinski et al., 2001; Lele and Ingber, 2006). GFP-tagged GEF-H1 and NMIIB had similar FRAP *t*_1/2_: 16±1.34 s and 17±1.01 s, respectively (mean±s.e.m.) (Fig. 4G), revealing that NMIIB possesses a similar protein turnover within FAs to that of GEF-H1 upon Dex treatment. Therefore, we focused further study on the mechanism of GEF-H1-dependent NMIIB association with FAs.

**Fig. 5.**
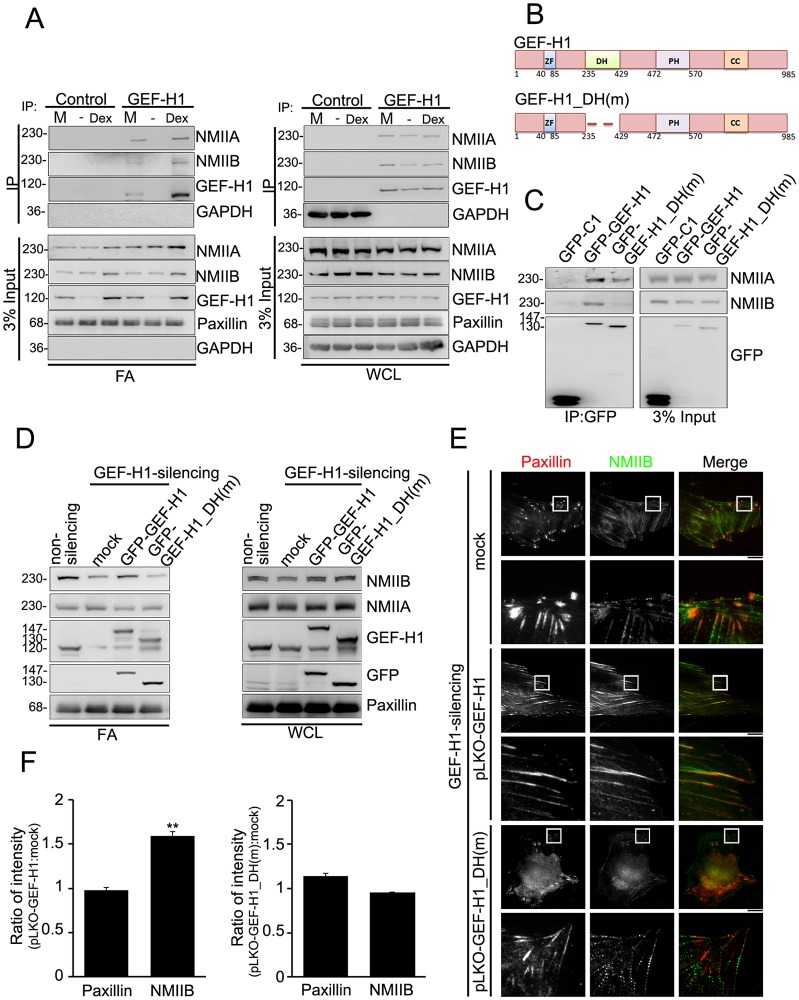
**GEF-H1 recruits NMIIB to FAs through its DH domain.** (A) FA fraction (FA) and whole-cell lysate (WCL) from MSC-3A6 cells treated with control culture medium (M) or serum-starved MSC-3A6 cells treated with ethanol (–) or Dex (0.1 µM) for 6 h was immunoprecipitated using the control (rabbit anti-GAPDH) or anti-GEF-H1 antibodies, and analyzed by western blotting. The 3% input of FA fraction was analyzed by western blotting. (B) Diagram of the domain structures of GEF-H1 and GEF-H1_DH(m). ZF, zinc-finger motif; DH, Dbl homology domain; PH, pleckstrin homology domain; CC, coiled-coil domain. (C) Whole-cell lysates from serum-starved MSC-3A6 cells expressing GFP–C1, GFP–GEF-H1 or GFP–GEF-H1_DH(m) treated with Dex (0.1 µM, 6 h) were immunoprecipitated using GFP-Trap beads. The immunoprecipitated complexes and the 3% input of whole-cell lysate were then analyzed by western blotting. (D) The FA fraction (FA) and the whole-cell lysate (WCL) from serum-starved non-silencing and GEF-H1-silencing MSC-3A6 cells expressing pGFP-C1 (mock), pGFP-GEF-H1 or pGFP-GEF-H1_DH(m) and treated with Dex (0.1 µM, 6 h) were analyzed by western blotting. (E) TIRF images of immunolocalized paxillin (red) and NMIIB (green) in GEF-H1-silencing MSCs expressing pLKO-vector (mock), pLKO-GEF-H1, or pLKO-GEF-H1_DH(m) and treated with Dex (0.1 µM, 6 h). Scale bars: 20 µm. The boxed 20 µm×20 µm areas indicated in the upper image rows are magnified in the row below. (F) Ratio of average density (intensity per µm^2^) of paxillin or NMIIB within segmented FAs of GEF-H1-silencing MSCs expressing pLKO-GEF-H1 relative to mock, or GEF-H1-silencing MSCs expressing pLKO-GEF-H1_DH(m) relative to mock (*n* = 11 cells for each condition). Data are mean±s.e.m. **P<0.005.

Our results showing that GEF-H1 localizes mostly in the FAs of MSCs upon treatment with Dex, and that GEF-H1 is required for the recruitment of NMIIB in Dex-stimulated FAs, suggests that GEF-H1 serves as a Dex-sensitive scaffold for FA recruitment of NMIIB. To verify this, we first examined the association of GEF-H1 with NMIIB in FAs with an immunoprecipitation assay in the FA fractions of MSC-3A6 cells. Fig. 5A revealed the association of NMIIB with GEF-H1 in FAs, concomitant with increase abundance of GEF-H1 and NMIIB in the FA fraction, upon Dex treatment. Surprisingly, NMIIA was also strongly co-precipitated by anti-GEF-H1 antibodies in the Dex-stimulated FAs (Fig. 5A), despite the finding that NMIIA in FAs did not seem to be affected by GEF-H1 expression (Fig. 4A,B). To determine whether the interactions between GEF-H1 and NMIIB or NMIIA were altered by Dex, we investigated their associations by immunoprecipitation of MSC-3A6 lysates. We found that the levels of NMIIB and NMIIA in the GEF-H1 immunoprecipitates were not changed regardless of Dex treatment (Fig. 5A). Taken together, GEF-H1 appears to interact with NMIIB and/or NMIIA in the cytoplasm and FAs, but only positively regulates the recruitment of NMIIB in FAs. It appears that NMIIB localizes mostly at FAs mainly through the GEF-H1–NMIIB interaction. Although GEF-H1 also interacts with NMIIA, this interaction is not crucial for NMIIA FA recruitment.

NMII is known to directly interact with the Dbl homology (DH) domain of several of the Dbl family of GEFs, including β-PIX, Tiam1 and Vav1 (Lee et al., 2010), implying that GEF-H1 might interact with NMII through its DH domain. To examine whether enrichment of NMIIB in Dex-stimulated FAs was dependent on the DH domain of GEF-H1, we first generated a GEF-H1 construct without the DH domain, which we termed GEF-H1_DH(m) (Fig. 5B), and assayed the association of the expressed protein with NMIIB in an immunoprecipitation assay. Immunoprecipitation was carried out using lysate from MSC-3A6 cells expressing pGFP-C1, pGFP-GEF-H1 or pGFP-GEF-H1_DH(m) with GFP-Traps beads and analyzed by immunoblotting. As shown in Fig. 5C, both NMIIB and NMIIA were found to be associated with GFP–GEF-H1, whereas neither protein was detectable in mock-transfected lysate. In the lysate of pGFP-GEF-H1_DH(m)-transfected cells, the co-precipitation of NMIIB was substantially reduced, whereas the level of NMIIA was not substantially changed (Fig. 5C). These results confirm that the DH domain of GEF-H1 serves as an important region for NMIIB association, but not for NMIIA interaction.

We then examined the effects of the DH domain of GEF-H1 on the recruitment of NMIIB in Dex-stimulated FAs. Immunoblotting of FA fractions from Dex-stimulated GEF-H1-silencing MSC-3A6 cells expressing pGFP-C1 (mock), pGFP-GEF-H1 or pGFP-GEF-H1_DH(m) revealed that the FA accumulation of NMIIB was substantially rescued by expressing pGFP-GEF-H1 but not pGFP-GEF-H1_DH(m) (Fig. 5D). To further examine the effects of GEF-H1_DH(m) on the enrichment of NMIIB in FAs of MSCs, we generated GEF-H1-silencing MSCs expressing pLKO vector (mock), pLKO-GEF-H1 or pLKO-GEF-H1_DH(m) using a lentivirus-based expression system (supplementary material Fig. S4A), and imaged the endogenous NMIIB and FA marker paxillin using TIRF microscopy analysis. Increased accumulation of NMIIB was observed in FAs of GEF-H1-silencing MSCs expressing GEF-H1, but not in those of cells expressing GEF-H1_DH(m) or mock (Fig. 5E). Quantification of the ratio of fluorescence density of paxillin and NMIIB in FAs indicated that mock, GEF-H1 or GEF-H1_DH(m) expression did not change the paxillin FA density. In contrast, the FA density of NMIIB was significantly increased (60%) by GEF-H1 expression and not GEF-H1_DH(m) expression, as compared with mock expression in GEF-H1-silencing MSCs (Fig. 5F). Thus, the DH domain of GEF-H1 plays a crucial role in recruiting NMIIB to Dex-stimulated FAs.

### FA recruitment of NMIIB regulates stress fiber polarization and FA formation

**Fig. 6.**
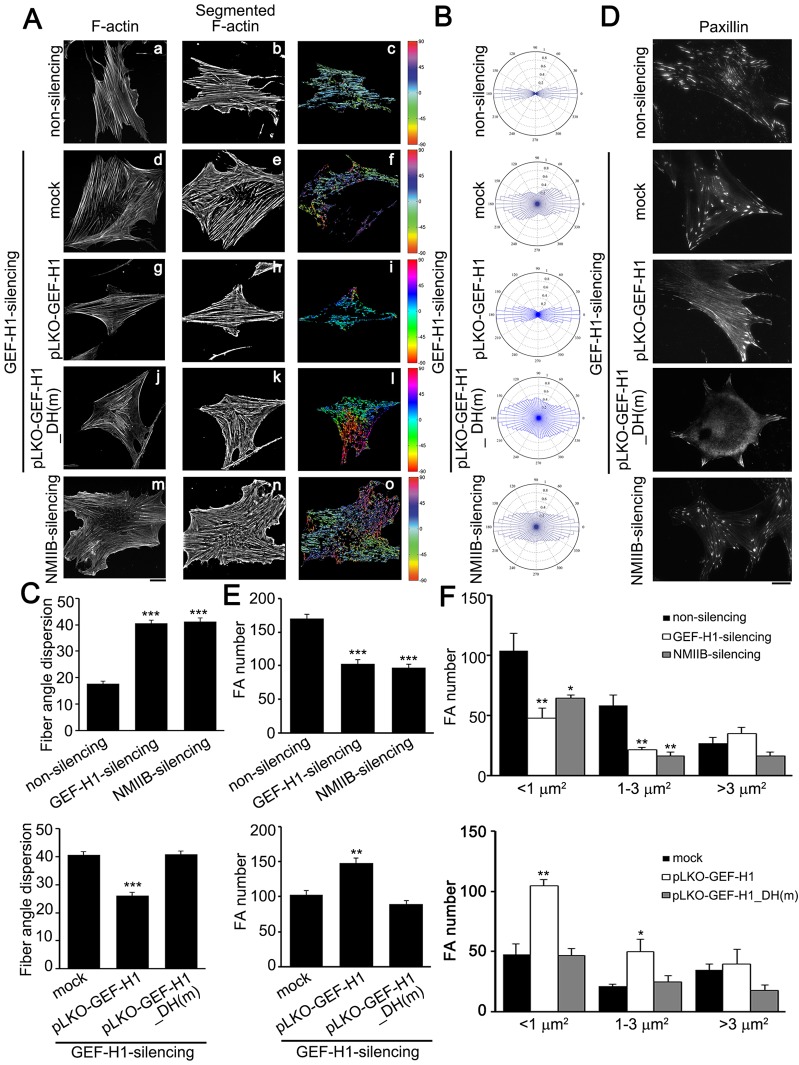
**GEF-H1-mediated FA recruitment of NMIIB controls stress fiber polarization and FA formation.** (A) Serum-starved non-silencing (a–c), GEF-H1-silencing MSCs expressing pLKO vector (mock) (d–f), pLKO-GEFH1 (g–i) or pLKO-GEF-H1_DH(m) (j–l), and NMIIB-silencing MSCs (m–o) were treated with Dex (0.1 µM, 6 h), and immunostained with FITC–phalloidin, to localize F-actin (a, d, g, j and m). Scale bar: 20 µm. After image segmentation, the image was rotated to set the median stress-fiber-orientation as horizontal (b, e, h, k and n). The orientation of each fiber was depicted with a specific color as indicated by the color at the right-hand side of each panel (c, f, i, l and o). (B,C) The polar histograms (B) and spread distribution (C) of stress fiber orientations were statistically calculated from non-silencing (*n* = 19 cells), or GEF-H1-silencing MSCs expressing pLKO vector (mock) (*n* = 18 cells), pLKO-GEFH1 (*n* = 15 cells) or pLKO-GEF-H1_DH(m) (*n* = 15 cells), and NMIIB-silencing MSCs (*n* = 14 cells). Data are mean±s.e.m. ***P<0.0001 (C, top: compared with non-silencing; bottom: compared with mock). (D) Serum-starved non-silencing, NMIIB-silencing, and GEF-H1-silencing MSCs expressing pLKO-vector (mock), pLKO-GEFH1 or pLKO-GEF-H1_DH(m) were treated with Dex (0.1 µM, 6 h), immunostained with paxillin, to visualize FAs, and imaged by TIRF microscopy. Scale bar: 20 µm. (E) The number of segmented paxillin-marked FAs of MSCs, as described in D. Data are mean±s.e.m. (non-silencing, *n* = 8 cells; NMIIB-silencing, *n* = 6 cells; GEF-H1-silencing cells expressing mock, *n* = 9 cells; expressing pLKO-GEF-H1, *n* = 7 cells; expressing pLKO-GEF-H1_DH(m), *n* = 6 cells). **P<0.005, ***P<0.0001 (top, compared with non-silencing; bottom, compared with mock). (F) Size distribution of segmented paxillin-marked FAs of MSCs, as described in D. Data are mean±s.e.m. (non-silencing, *n* = 8 cells and 1506 FAs; NMIIB-silencing, *n* = 6 cells and 581 FAs; GEF-H1-silencing expressing mock, *n* = 9 cells and 835 FAs; expressing pLKO-GEF-H1, *n* = 7 cells and 1358 FAs; expressing pLKO-GEF-H1_DH(m), *n* = 6 cells and 537 FAs). *P<0.05; **P<0.005 (top, compared with non-silencing; bottom, compared with mock).

We then examined FA recruitment of NMIIB-controlled polarized stress fibers upon Dex treatment. We generated non-silencing, NMIIB-silencing (supplementary material Fig. S4B) and GEF-H1-silencing MSCs expressed using pLKO-vector (mock), pLKO-GEF-H1 or pLKO-GEF-H1_DH(m), respectively (supplementary material Fig. S4A), and measured the orientation of stress fibers and fiber angle dispersion by analyzing the images of F-actin (Fig. 6A) as described above. Fig. 6B,C shows that NMIIB-silencing MSCs exhibited wider distributions of stress fiber orientations than non-silencing MSCs, similar to that of mock or pLKO-GEF-H1_DH(m) expression in GEF-H1-silencing MSCs. In addition, the disruption of stress fiber alignment in GEF-H1-silencing MSCs was rescued by expression of pLKO-GEF-H1. Supplementary material Fig. S4B showed that silencing of NMIIB did not change the expression of GEF-H1 and NMIIA (Raab et al., 2012), implying that the effects of NMIIB on stress fiber polarization are not due to the decrease in NMIIA or GEF-H1. These findings indicate that GEF-H1-dependent FA recruitment of NMIIB regulates the Dex-induced anisotropic orientation of stress fibers in MSCs.

We next examined the role of NMIIB in Dex-induced FA formation. Immunolocalization of paxillin in non-silencing, NMIIB-silencing and GEF-H1-silencing MSCs, expressing pLKO-vector (mock), pLKO-GEF-H1, and pLKO-GEF-H1_DH(m), respectively, upon Dex treatment showed that silencing of NMIIB significantly decreased total FA number of MSCs (Fig. 6D,E), especially that of medium-sized FAs (1∼3 µm^2^) and small sized FAs (<1 µm^2^) (Fig. 6F), similar to GEF-H1-silencing MSCs (Fig. 6D–F). The effects of GEF-H1-silencing MSCs on FA number and FA size distribution were rescued by pLKO-GEF-H1 but not by pLKO-GEF-H1_DH(m), expression (Fig. 6D–F). These results indicate that GEF-H1-mediated accumulation of NMIIB in FAs is required for the modulation of FA formation in MSCs upon Dex treatment.

### FA recruitment of NMIIB regulates cytoskeletal mechanics

**Fig. 7.**
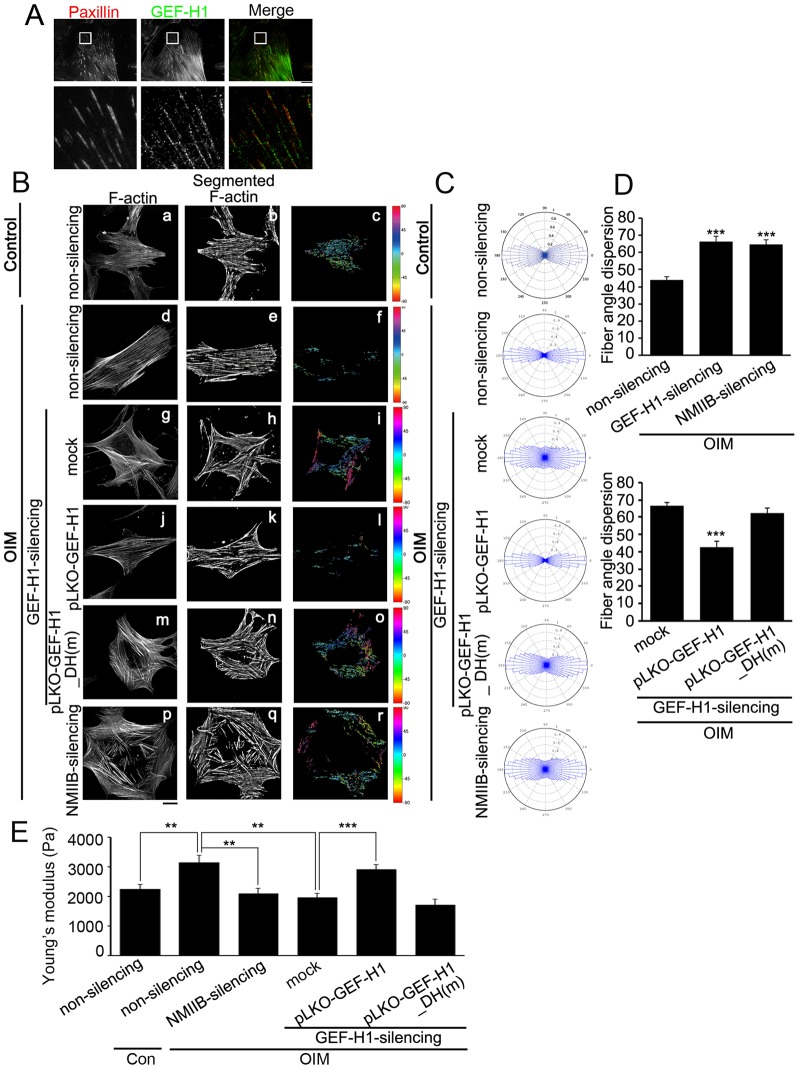
**GEF-H1-mediated FA recruitment of NMIIB controls cytoskeletal mechanics.** (A) TIRF images of immunolocalized paxillin (red) and GEF-H1 (green) in MSCs treated with OIM for 48 h. Scale bar: 20 µm. The boxed 20 µm×20 µm areas indicated in the top images are magnified in the images below. (B) Non-silencing MSCs treated with control culture medium (a–c), non-silencing MSCs (d–f), GEF-H1-silencing MSCs expressing pLKO vector (mock) (g–i), pLKO-GEFH1 (j-l) or pLKO-GEF-H1_DH(m) (m–o), and NMIIB-silencing MSCs (p–r) were treated with OIM for 48 h, and immunostained with FITC–phalloidin, to localize F-actin (a, d, g, j, m and p). Scale bar: 20 µm. After image segmentation, the image was rotated to set the median stress-fiber-orientation as horizontal (b, e, h, k, n and q). The orientation of each fiber was depicted with a specific color as indicated by the color at the right-hand side of each panel (c, f, i, l, o and r). (C,D) The polar histograms (C) and spread distribution (D) of stress fiber orientations were statistically calculated (*n* = 15 cells for each conditions). Data are mean±s.e.m. ***P<0.0001 (D, top, compared with non-silencing; bottom, compared with mock). (E) Stiffness (Young's modulus; Pa) of non-silencing MSCs treated with control culture medium (*n* = 49 cells) and non-silencing (*n* = 50 cells), NMIIB-silencing (*n* = 33 cells) and GEF-H1-silencing MSCs expressing pLKO-vector (mock) (*n* = 39 cells), pLKO-GEFH1 (*n* = 35 cells) or pLKO-GEF-H1_DH(m) (*n* = 48 cells) were treated with OIM for 48 h. Data are mean±s.e.m. **P<0.005, ***P<0.0001.

The dependence of Dex-mediated stress fiber polarization on FA recruitment of NMIIB raises a possibility that NMIIB in FAs might regulate the orientation of stress fiber and cytoskeletal mechanics under the osteogenic differentiation conditions. As GEF-H1 is the central regulator of the FA abundance of NMIIB, we first confirmed FA localization of GEF-H1 in MSCs cultured for 48 h in OIM. Immunolocalization of paxillin and GEF-H1 showed that GEF-H1 was localized in FAs (Fig. 7A). Visualization of NMIIB in MSCs revealed that, in cells expressing the non-silencing shRNAs, NMIIB localized in FAs upon OIM treatment, but that this localization was suppressed by GEF-H1 knockdown (supplementary material Fig. S4C,Da). This inhibitory effect was rescued by expressing pLKO-GEF-H1 (supplementary material Fig. S4C,Db), but not pLKO-GEF-H1_DH(m) (supplementary material Fig. S4C,Dc). Thus, under the osteogenic differentiation conditions, GEF-H1 is enriched in FAs to mediate the recruitment of NMIIB through its DH domain.

To calculate the orientation of stress fibers, we analyzed the images of F-actin as described above and showed that non-silencing MSCs had a higher degree of alignment of stress fiber orientation upon OIM treatment. Cells with silencing of NMIIB exhibited a wider distribution of stress fiber orientation, as was apparent in GEF-H1-silencing MSCs expressing pLKO-vector (mock) or pLKO-GEF-H1_DH(m) (Fig. 7B–D). Taken together, these results confirm that upon osteogentic induction, the FA localization of GEF-H1 recruits NMIIB into FAs that play a crucial role in anisotropic orientation of stress fibers in MSCs.

As the orientation of stress fibers, together with the dynamic FAs, has been linked to the spatial organization of intracellular tension and changes in cell shape (Lee et al., 2013; Zemel et al., 2010a; Zemel et al., 2010b), we next investigated whether cell stiffness (viscos-elasticity) was changed due to the increased abundance of NMIIB in FAs. The non-silencing, NMIIB-silencing and GEF-H1-silencing MSCs, expressing pLKO-vector (mock), pLKO-GEF-H1 or pLKO-GEF-H1_DH(m), respectively, were cultured in control culture medium or OIM for 48 h and their Young's modulus (Pa) was measured using atomic force microscopy (AFM). We found that the stiffness of non-silencing MSCs was significantly higher when they were cultured in OIM than in control culture medium (Fig. 7E). Furthermore, under the osteogenic differentiation condition, silencing of NMIIB or GEF-H1 significantly softened the cells, as compared to the non-silencing MSCs. The cell stiffness in GEF-H1-silencing MSCs was increased by pLKO-GEF-H1 expression, but not by pLKO-GEF-H1_DH(m) expression (Fig. 7E). Taken together, these findings indicate that the recruitment of NMIIB in FAs affects cytoskeletal mechanics, leading to the modulation of MSC stiffness.

### GEF-H1 regulates the MSC commitment through NMIIB-mediated cytoskeletal mechanics

**Fig. 8.**
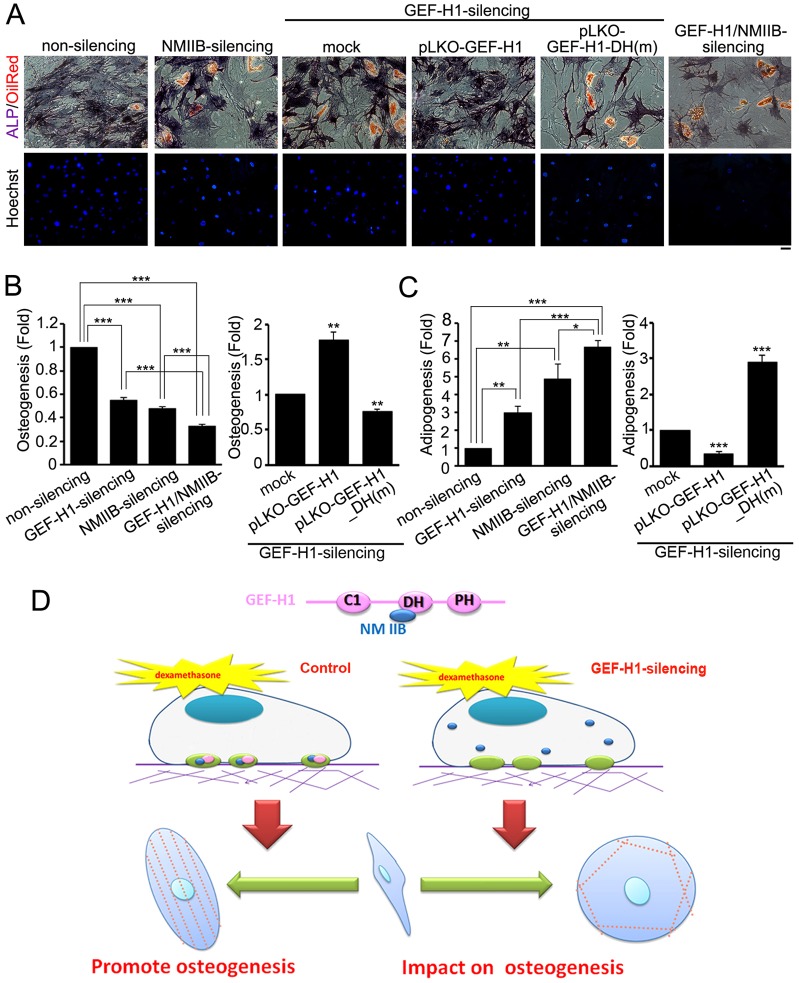
**MSCs adipogenesis versus osteogenesis depends on FA recruitment of NMIIB.** (A) Non-silencing, NMIIB-silencing, GEF-H1 and NMIIB-silencing, and GEF-H1-silencing MSCs expressing pLKO-vector (mock), pLKO-GEFH1 or pLKO-GEF-H1_DH(m) were treated with mixed differentiation medium (OIM ∶ AIM = 1∶1) for 14 days, and stained for the presence of lipid (Oil Red O; red), the activity of ALP (purple) and Hoechst 33342 (blue). Scale bar: 50 µm. (B) Fold change in cells showing osteogenesis (ALP activity) and (C) adipogenesis (Oil red O level) in GEF-H1-silencing (*n* = 348 cells), NMIIB-silencing (*n* = 408 cells) or GEF-H1- and NMIIB-silencing (*n* = 370 cells), normalized to non-silencing MSCs (*n* = 571 cells), and GEF-H1-silencing MSCs expressing pLKO-GEFH1 (*n* = 596 cells) or pLKO-GEF-H1_DH(m) (*n* = 212 cells), normalized to pLKO-vector (mock) (*n* = 348 cells). Data are mean±s.e.m. **P<0.005, ***P<0.0001. (D) Model of FA-mediated signaling in the control of MSC commitment to osteogenesis. The FA abundance of GEF-H1 acts as a scaffold protein to recruit NMIIB, driving anisotropic orientation of stress fibers, FA organization and MSC osteoblast differentiation. Interference with the abundance of NMIIB in FAs alters actin cytoskeletal organization, cell stiffness, and MSC commitment to adipogenesis. The GEF-H1- and NMIIB-mediated FA signaling appears necessary to reorganize cytoskeletal mechanics, leading to an osteogenic–adipogenic switch in MSC lineage commitment.

As the high and low levels expression of GEF-H1 positively correlates to osteogenic and adipogenic differentiation, respectively, we investigated whether the effect on MSC lineage commitment is due to FA recruitment of NMIIB. We cultured the non-silencing, NMIIB-silencing, GEF-H1/NMIIB-silencing and GEF-H1-silencing MSCs, expressing pLKO-vector (mock), pLKO-GEF-H1 or pLKO-GEF-H1_DH(m), respectively, in a mixed differentiation medium (OIM∶AIM = 1∶1) for 14 days. ALP or lipid droplet staining revealed that silencing of NMIIB or GEF-H1 increased adipogenesis and decreased osteogenesis (Fig. 8A–C). Silencing of both GEF-H1 and NMIIB further decreased osteogenesis and increased adipogenesis, in comparison with GEF-H1-silencing or NMIIB-silencing MSCs (Fig. 8A–C). The shift in lineages in GEF-H1-silencing MSCs was rescued by pLKO-GEF-H1 expression, but not pLKO-GEF-H1_DH(m) expression (Fig. 8A–C). The examination of stress fiber orientation in MSCs cultured in OIM for 14 days (data not shown) showed similar results as seen in Fig. 7B–D. Taken together, these findings suggest that, although GEF-H1-mediated FA signaling is downstream of differentiation stimuli, the control of the osteogenic-to-adipogenic commitment switch mediated by stress fiber polarization is regulated through FA recruitment of NMIIB.

## DISCUSSION

Our study profiled GEF-H1-dependent FA composition changes and uncovered a new role of GEF-H1 in regulating cytoskeletal architecture and MSC osteogenic differentiation through FA signaling. To further understand the role of GEF-H1 in FAs, we searched for proteins recruited to FAs in a GEF-H1-dependent manner and examined the effect of their association. We focused on the GEF-H1-dependent recruitment of NMIIB to FAs, showing that it controlled the anisotropic orientation of stress fibers, the stiffness of cells and commitment of MSCs to osteogenic fate (Fig. 8D). Here, we demonstrated that MSC commitment was regulated through FA-mediated signaling, which is crucial in the control of cytoskeletal mechanics and the stiffness of cells.

Our results revealed for the first time the association of GEF-H1 (Fig. 3), NMIIA and NMIIB (Fig. 4) in FAs, although GEF-H1, NMIIA and NMIIB had not been previously listed in the integrin adhesome (Zaidel-Bar and Geiger, 2010; Zaidel-Bar et al., 2007). FA accumulation of NMIIB was confirmed to be regulated by binding with GEF-H1 (Figs 4 and 5). However, GEF-H1 did not control the accumulation of NMIIA at FAs, although NMIIA had been demonstrated to interact with GEF-H1 (Fig. 5A,C) (Lee et al., 2010). By assessing the Dex sensitivity of the GEF-H1–NMIIA and GEF-H1–NMIIB interactions, we showed that these interactions were not influenced by signaling induced by Dex (Fig. 5A), implying that NMIIA is recruited to FAs through other new interacting proteins that are yet to be identified. Nevertheless, our results showed that the abundance of GEF-H1 in FAs was sensitive to Dex (Fig. 3), supporting the notion that GEF-H1 served as a crucial scaffold protein in the recruitment of NMIIB to FAs of MSCs under osteogenic conditions.

Our study also revealed a previously unrecognized role of an interaction between GEF-H1 and NMIIB at FAs that takes place in the physiologically relevant context of stress fiber polarization in MSC osteogenic differentiation. We showed that FA recruitment of NMIIB, through GEF-H1, facilitated the directional orientation of stress fibers and FA formation. In OIM-stimulated GEF-H1-silencing MSCs, we demonstrated that GEF-H1, but not GEF-H1_DH(m) (a GEF-H1 mutant with a deletion of the DH domain), rescued defects in stress fiber polarization, leading to a closer alignment of the stress fibers with the major axis of the cells (Fig. 7B–D). This observation specifies a new function of the GEF-H1 DH domain, which, through its association with NMIIB, targets NMIIB for FA localization; this, in turn, mediates polarization and/or alignment of stress fibers within the cell. The anisotropic orientation of stress fibers, together with the dynamic FAs, has been linked to the spatial organization of intracellular forces and changes in cell morphology, leading to the reinforcement of cell tension (Lee et al., 2013; Zemel et al., 2010a; Zemel et al., 2010b). The structural and mechanical polarization of the cytoskeleton has been observed in cells responding to diverse types of active mechanical stimuli (del Alamo et al., 2008; Hur et al., 2012; Kaunas et al., 2005). It is thus conceivable that the recruitment of NMIIB, through the DH domain of GEF-H1, to FAs contributes to a polarized distribution of stress fibers and FA formation, thereby generating an increase in intracellular tension and modulating cell shape. Indeed, FA recruitment of NMIIB, through GEF-H1, mediates the stiffness of MSCs (Fig. 7E), supporting the notion that the increased accumulation of the GEF-H1–NMIIB complexes in FAs might contribute to polarize the cellular tension along the stress fibers parallel to the long axis of the cells to commit indispensable events of osteogenesis, including cell shape, cell–matrix array and cell–cell alignment (Guilak et al., 2009; Müller et al., 2013; Rodríguez et al., 2004). It is known that ∼65% of the NMIIB-null mice die prior to birth due to defective heart and brain development, whereas some are born suffering from cardiac failure and die during the first day of life. Therefore, most studies focused on the role of NMIIB on the development of brain and heart (Takeda et al., 2003; Tullio et al., 1997). Although the studies of NMIIB-null mice do not mention the effects on bone mass, the size of newborn NMIIB-null mice is smaller than control mice (Tullio et al., 1997), implying NMIIB might contribute to bone strength and bone formation.

Our results, together with those from previous studies, support the notion that the regulation of actin cytoskeleton by microtubule dynamics can be mediated by GEF-H1 (Chang et al., 2008; Enomoto, 1996; Krendel et al., 2002), which is in turn regulated by an interaction with polymerized microtubules (Krendel et al., 2002). Indeed, we showed that drug-induced microtubule stabilization (taxol) resulted in changes in FA organization, as well as orientation of contractile stress fibers, similar to the changes induced by silencing of GEF-H1 (supplementary material Fig. S1D; Fig. 2D–F). GEF-H1 has been identified as a GEF that activates RhoA through its DH domain (Ren et al., 1998), which is known to promote myosin-II-driven contractile force and stress fiber formation. However, we showed that silencing of GEF-H1 or expression of GEF-H1_DH(m) did not abolish the formation of stress fibers induced by Dex or OIM (Fig. 6A; Fig. 7B), indicating that the GEF activity of GEF-H1 was not required for stress fiber formation upon osteogenic induction. It also suggests that, upon osteogenic induction, GEF-H1 is not responsible for the regulation of RhoA activity on myosin II contractility (phosphorylation at Thr18/Ser19 of MLC) and stress fiber formation, implying the existence of another RhoA GEF that mediates RhoA activation. To date, a few members of the Rho GEF family, for example, leukemia-associated Rho GEF (LARG, also known as Rho guanine nucleotide exchange factor 12, encoded by *ARHGEF12*) and p115-RhoGEF (also known as Rho guanine nucleotide exchange factor, encoded by *ARHGEF1*), have been examined and shown to increase the activity of RhoA (Guilluy et al., 2011), thus playing a pivotal role in RhoA signaling in response to Dex. Given that drug-induced microtubule depolymerization (nocodazole)-induced stress fiber formation was suppressed by silencing of GEF-H1 (Fig. 2C), the amount of unbound GEF-H1 might determine its contribution to Rho-dependent regulation of the actin cytoskeleton in MSCs. In addition, the cellular distribution of GEF-H1 might lead to its specific biological functions. For examples, microtubule-bound GEF-H1 could contribute to the stabilization of microtubules, cytosolic GEF-H1 might serve as a GEF for RhoA activation (Krendel et al., 2002) and FA-accumulated GEF-H1 could mediate FA organization and stress fiber polarization. As Dex begins to exert its activity on FA maturation, the increased abundance of GEF-H1 in FAs promotes the enrichment of GEF-H1–NMIIB complex to affect the organization of FAs, actin stress fibers and cell stiffness.

Several crucial questions remain about Dex-induced recruitment of GEF-H1 to FAs. Of the 41 well-known GEF-H1-interacting proteins, only RhoA and Rac1 are well-characterized FA proteins (Zaidel-Bar and Geiger, 2010; Zaidel-Bar et al., 2007). Although NMIIA, NMIIB, VPC, 14-3-3τ and 14-3-3ζ are reproducibly identified in the FA fractions, they are not listed in the integrin adhesome (Zaidel-Bar and Geiger, 2010; Zaidel-Bar et al., 2007). Therefore, the recruitment of GEF-H1 to FAs might be mediated through its interaction with one or more of the well-known FA proteins, or through other novel interacting proteins yet to be identified. Additionally, the mechanisms for NMIIB FA recruitment that promote stress fiber polarization remain unknown. Future studies are clearly needed to help clarify these important questions.

## MATERIALS AND METHODS

### Cells

Human mesenchymal stem cells (MSCs) (Lonza) stably expressing shRNA or pLKO-AS3W-related expression constructs were generated using a lentiviral shRNA system according to the manufacturer's instructions. Transient transfections were performed by nucleofection (Lonza) with the Human MSC Nucleofector Kit and program U-23. The MSC-3A6 cells (a gift from Shih-Chieh Hung, National Yang-Ming University, Taiwan), derived from MSCs (Hung et al., 2004; Tsai et al., 2010), had stem-like properties and possessed a longer life span in culture, so these cells were used to reach the desired cell number for all of the biochemical experiments. Lipofectamine 2000 was used for transfection. For all experiments, cells were seeded on 10 µg/ml fibronectin-coated coverslips or plates.

### Plasmids and reagents

To derive the GFP–C1-GEF-H1 (with shRNA resistance), full-length GEF-H1 cDNA was PCR amplified from the template pCMV5-EGFP-GEF-H1, and cloned into pGFP-C1 (Clontech) (*Hin*dIII/*Kpn*I). Site-directed mutagenesis was then carried out to mutate two nucleotides of GEF-H1 (without altering the amino acid sequence) on the target sequence of GEF-H1 shRNA. For GFP–C1-GEF-H1_DH(m) (with shRNA resistance), amino acids 1–235 and 429–985 of GEF-H1 were amplified, and cloned into pGFP-C1 (*Hin*dIII/*Kpn*I/NcoI). Site-directed mutagenesis was again used to generate the pGFP-GEF-H1_DH(m) (with shRNA resistance) for all the experiments. For pLKO-AS3W-GEF-H1 and pLKO-AS3W-GEF-H1_DH(m), GEF-H1 and GEF-H1_DH(m) were amplified from pGFP-C1-GEF-H1 (with shRNA resistance) and pGFP-C1-GEF-H1_DH(m) (with shRNA resistance), respectively, and cloned into the pLKO-AS3W (National RNAi Core Facility Platform) (*Nhe*I/*Eco*RI). Dexamethasone and nocodazole were from Sigma, and taxol was from Millipore. Details of siRNA, shRNA and primer sequences, and the antibodies for western blotting and immunofluorescence are given in supplementary material Table S4.

### Immunofluorescence staining and image analysis

Immunofluorescence analyses were carried out as described previously (Betapudi et al., 2006). The relative abundance of NMIIB, GEF-H1, and paxillin in FAs was determined using Metamorph, as described previously (Kuo et al., 2011). The orientation of the stress fibers was determined in a similar manner to that described previously (Karlon et al., 1999) using an automated image analysis algorithm written in MATLAB.

### Fluorescence recovery after photobleaching

MSCs transiently expressed mApple–paxillin, to visualize FAs, and GFP–GEF-H1 or GFP–NMIIB. FRAP of GFP-tagged proteins was performed using a 100× 1.49NA Plan objective lens on the *iLas* multi-modal of TIRF (Roper)/spinning disk confocal (CSUX1, Yokogawa) microscope system. The 488-nm laser was used to photobleach the spot onto a single fluorescent FA. Images were acquired at intervals of 1 s before and after photobleaching using a Coolsnap HQ2 CCD (Photometrics). Image frequency was adjusted depending on the fluorescence photobleaching recovery rate of the GFP-tagged protein being imaged.

### Microscopy

Images were obtained using an *iLas* multi-modal of TIRF (Roper)/spinning disk confocal (CSUX1, Yokogawa) microscope system equipped with 40× 0.75NA, 60× 1.40NA or 100× 1.49NA Plan objective lenses (Nikon). Confocal images were captured using an EMCCD (ProEM, Princeton); TIRF images were obtained using 60× 1.40NA or 100× 1.49NA Plan objective lens with an ∼100-nm evanescent field depth on a Coolsnap HQ2 CCD (Photometrics).

### Measurement of cell stiffness by AFM indentation

Cells were plated at the density of 3×10^3^ cells/cm^2^, exposed to OIM for 48 h, and the stiffness of a cell was measured with the BioCell device (JPK Instruments, Berlin, Germany) (Chiou et al., 2013) on the JPK NanoWizard II AFM system (Costa, 2004; Li et al., 2008).

### MSC differentiation

The protocol of MSC differentiation was carried out as described previously (Yu et al., 2011). Only early passage MSCs were used for experimental studies. The osteogenesis induction medium (OIM) contained 0.1 µM Dex, 10 mM β-glycerolphosphate, 50 µM ascorbic acid-2-phosphate in control medium (DMEM containing 10% FBS). The adipogenesis induction medium (AIM) contained 1 µM Dex, 0.5 mM methtlisobutylxathine, 200 µM indomethacin, 10 µg/ml insulin in control medium (DMEM containing 10% FBS). Mixed differentiation medium contained 1∶1 OIM∶AIM (Kilian et al., 2010; McBeath et al., 2004).

### MSC staining

The protocol of MSC staining was carried out as described previously (McBeath et al., 2004). Cells were fixed in 4% paraformaldehyde, rinsed in PBS and then stained with Fast BCIP/NBT (Sigma) for the activity of alkaline phosphatase (ALP). To stain lipid, cells were rinsed in 60% isopropanol, stained with 30 mg/ml Oil Red O (Sigma) in 60% isopropanol, and rinsed in PBS. Cells were then stained with Hoechst 33342 in PBS to obtain the total cell count. Cells were photographed and counted using a Nikon Eclipse TE200.

### Statistical analysis

Statistical significance was measured by a two-tailed Student's *t*-test.

## Supplementary Material

Supplementary Material
