# Supplementary material for: GEF-H1 controls focal adhesion signaling that regulates mesenchymal stem cell lineage commitment
Source: J Cell Sci. 2014 Oct 1;127(19):4186–200. doi: 10.1242/jcs.150227 (PMC4179489; doi:10.1242/jcs.150227)
Supplement: Supplementary Material [file supp_127_19_4186__index.html]

Supplementary Material 

# GEF-H1 controls focal adhesion signaling that regulates mesenchymal stem cell lineage commitment

## JCS150227 Supplementary Material

**Files in this Data Supplement:**

- **Supplementary Material**
