# Supplementary material for: GEF-H1 controls focal adhesion signaling that regulates mesenchymal stem cell lineage commitment
Source: J Cell Sci. 2014 Oct 1;127(19):4186–200. doi: 10.1242/jcs.150227 (PMC4179489; doi:10.1242/jcs.150227)
Supplement: Supplementary Material [file supp_jcs.150227_JCS150227.pdf]

Huang_Fig. S1

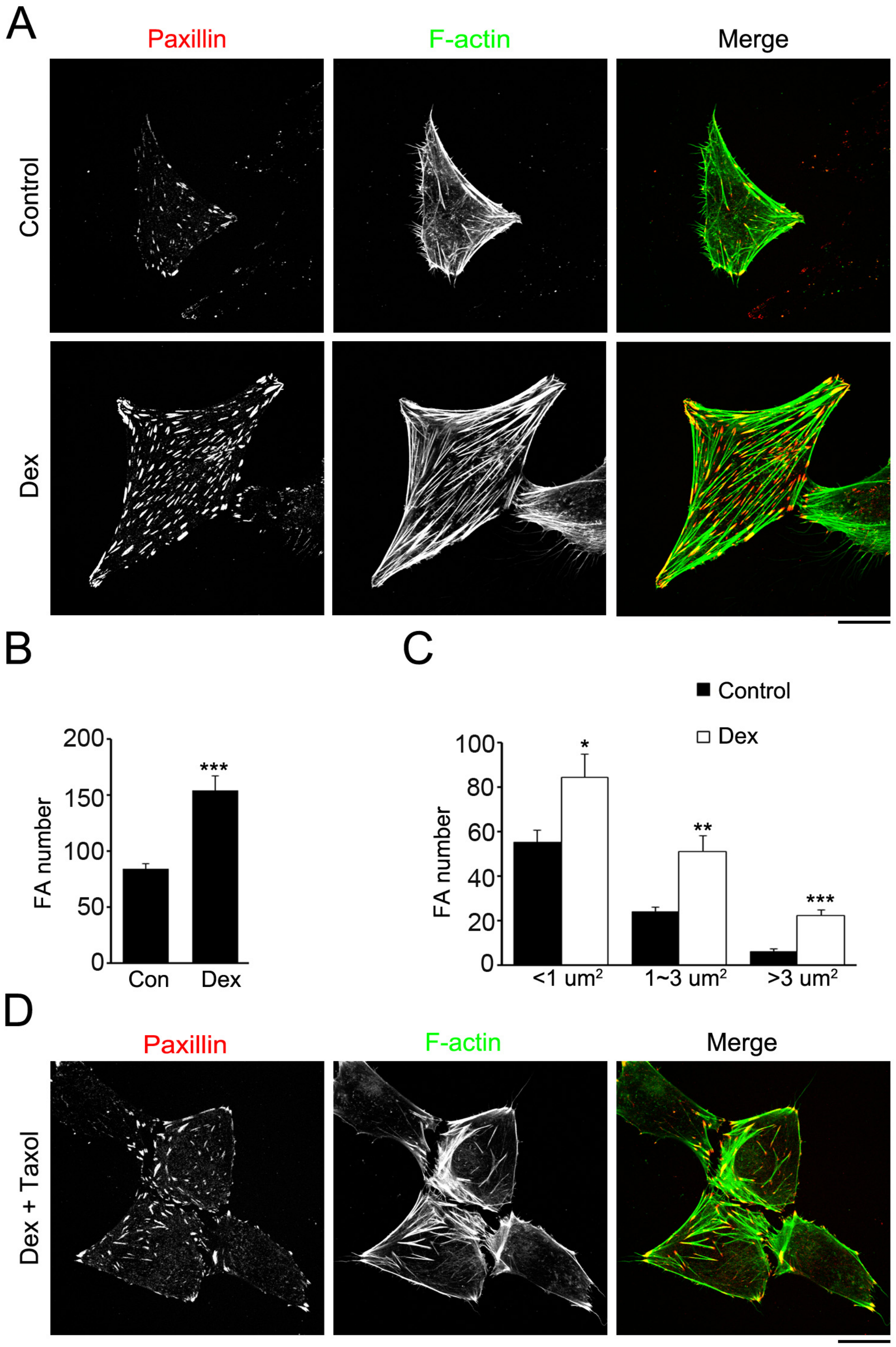
A
Paxillin
F-actin
Merge
Control
Dex
B
FA number
200
150
100
50
0
***
Con
Dex
C
Control
Dex
FA number
100
80
60
40
20
0
*
**
***
<1 um2
1~3 um2
>3 um2
D
Paxillin
F-actin
Merge
Dex + Taxol

Huang_Fig. S2

A

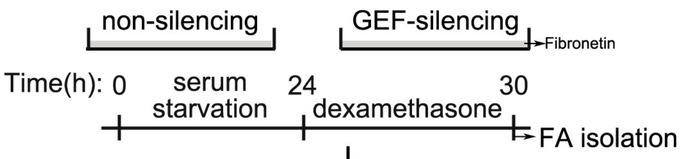


**FA isolation**

(1) remove culture medium and wash

(2) 3 mins hypotonic shock (2.5 mM triethanolame buffer)

(3) collect isolated FAs in RIPA buffer

(4) acetone precipitation

**Protein identification by proteomic analysis**

(1) reduction and alkylation

(2) trypsin digestion

(3) LC/MS-MS analysis (LTQ Orbitrap-XL) of peptides

(4) protein identification by Mascot software

**Protein quantification in the reproducible list**

(1) assemble the reproducible list (presence in ≧2 replicate runs)

(2) normalize the spectrum counts of individual protein in the reproducible list

**Normalization between experimental runs**

$$\frac{\text{spectrum counts of individual protein}}{\text{spectrum counts of all proteins}} \times 1000$$

**Normalization between experimental conditions**

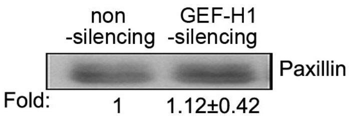


$$\text{Spetrum counts of individual protein in GEF-H1-silencing FA fraction} \times \frac{\text{spectrum counts of paxillin in control FA fraction}}{\text{spectrum counts of paxillin in GEF-H1-silencing FA fraction}}$$

(3) assemble the GEF-H1-modulated FA proteome

$$\text{Ratio of protein abundance} = \frac{\text{Normalized spectrum counts in control FA fraction}}{\text{Normalized spectrum counts in GEF-H1-silencing FA fraction}}$$

B

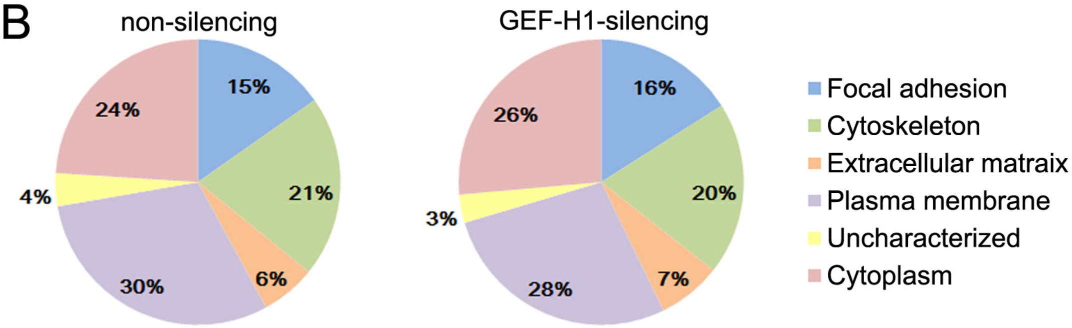

Huang_Fig. S3

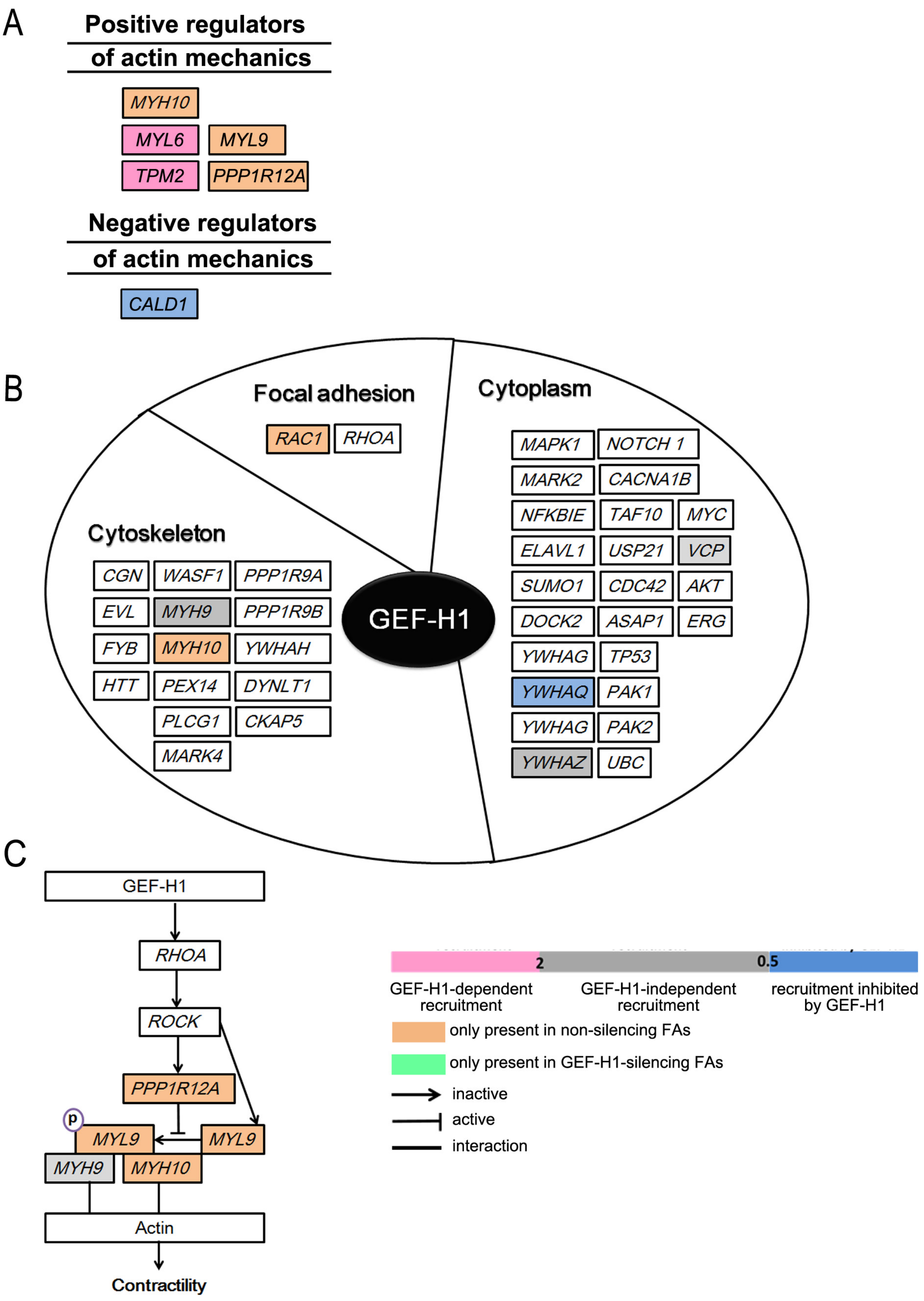
A
Positive regulators
of actin mechanics
MYH10
MYL6
MYL9
TPM2
PPP1R12A
Negative regulators
of actin mechanics
CALD1
B
Focal adhesion
RAC1
RHOA
Cytoplasm
MAPK1
NOTCH 1
MARK2
CACNA1B
NFKBIE
TAF10
MYC
ELAVL1
USP21
VCP
SUMO1
CDC42
AKT
DOCK2
ASAP1
ERG
YWHAG
TP53
YWHAQ
PAK1
YWHAG
PAK2
YWHAZ
UBC
Cytoskeleton
CGN
WASF1
PPP1R9A
EVL
MYH9
PPP1R9B
FYB
MYH10
YWHAH
HTT
PEX14
DYNLT1
PLCG1
CKAP5
MARK4
GEF-H1
C
GEF-H1
RHOA
ROCK
PPP1R12A
p
MYL9
MYL9
MYH9
MYH10
Actin
Contractility
2
0.5
GEF-H1-dependent recruitment
GEF-H1-independent recruitment
recruitment inhibited by GEF-H1
only present in non-silencing FAs
only present in GEF-H1-silencing FAs
inactive
active
interaction

Huang_Fig. S4

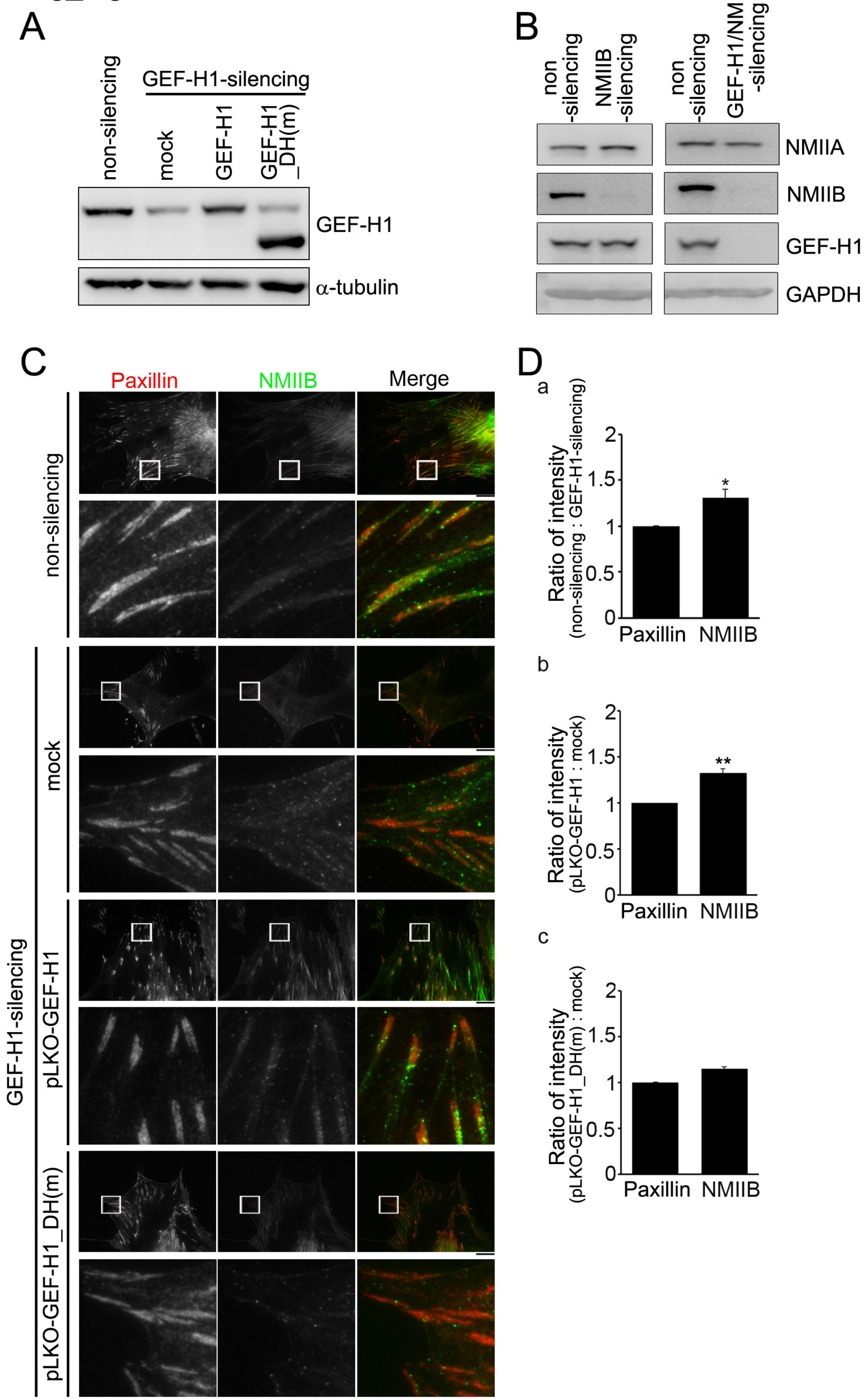
A
non-silencing
GEF-H1-silencing
mock
GEF-H1
GEF-H1 _DH(m)
GEF-H1
α-tubulin
B
non -silencing
NMIIB -silencing
non -silencing
GEF-H1/NMIIB -silencing
NMIIA
NMIIB
GEF-H1
GAPDH
C
Paxillin
NMIIB
Merge
non-silencing
mock
GEF-H1-silencing
pLKO-GEF-H1
pLKO-GEF-H1_DH(m)
D
a
Ratio of intensity (non-silencing : GEF-H1-silencing)
2
1.5
1
0.5
0
*
Paxillin
NMIIB
b
Ratio of intensity (pLKO-GEF-H1 : mock)
2
1.5
1
0.5
0
**
Paxillin
NMIIB
c
Ratio of intensity (pLKO-GEF-H1_DH(m) : mock)
2
1.5
1
0.5
0
Paxillin
NMIIB

**Huang_Supplemental figure legends**

**Fig. S1. Effects of microtubule stabilization on Dex-induced stress fiber formation and FA maturation.**

(A) Serum-starved MSCs treated with ethanol (control) or Dex (0.1μM, 6h) were immunostained with FITC-phalloidin, to localize F-actin (green), and paxillin, to localize FAs (red). Bar, 20 μm. (B) The number of segmented paxillin-marked FAs of MSCs, as described in (A). Data are mean±s.e.m (Con, n=25 cells; Dex, n=22 cells). ***p<0.0001 (compared with control). (C) Size distribution of segmented paxillin-marked FAs of MSCs, as described in (A). Data are mean±s.e.m. (Control, n=25 cells/2354 FAs; Dex, n=22 cells/3440 FAs). *p<0.05; **p<0.005; ***p<0.0001 (compared with control). (D) Serum-starved MSCs treated with Dex (0.1μM) and taxol (20μM) for 6h were immunostained with FITC-phalloidin, to localize F-actin (green), and paxillin, to localize FAs (red). Bar, 20 μm.

**Fig. S2. A quantitative proteomic workflow for the GEF-H1-regulated FA proteome.**

(A) Flow diagram of the procedure to isolate and prepare FAs from non-silencing and GEF-H1-silencing MSC-3A6 cells for protein identification by proteomic analysis, and for calculating the changes in abundance of identified proteins in FAs in response to GEF-H1 expression. In detail, Serum-starved non-silencing and GEF-H1-silencing MSC-3A6 cells were treated with Dex (0.1μM) for 6h. FAs were then isolated, collected, precipitated, solubilized, reduced, alkylated, and trypsin digested. The resulting peptides were analyzed by one-dimensional LC-MS/MS. Tandem mass spectra were generated and searched against those peptides derived from proteins in a human protein database using the MASCOT program. Each protein was identified by one or more peptides, with the false positive identification rate set at ≤ 1%. To derive the reproducible lists from non-silencing and GEF-H1-silencing FA fractions, only proteins that were reproducibly identified at least two out of five replicate runs (five independent experimental runs for each condition) were included. To quantify the relative levels of individual FA protein isolated from non-silencing and GEF-H1-silencing MSC-3A6 cells and assemble the GEF-H1-modulated FA proteome, the spectrum counts of individual protein (the number of peptides matched to each identified protein during mass spectrometric database search) was used as a rough estimate of the abundance of that protein in a complex mixture. Before further calculation,

the raw spectrum count of each protein was normalized to correct the variations between experimental runs and experimental conditions. To correct for run-to-run variation, the raw spectrum count of a protein was divided by the total spectrum count of the run and multiplied by 1000 to obtain a readable number. Subsequently, variation resulting from sample preparation due to experimental conditions (isolated FA fractions of non-silencing versus GEF-H1-silencing MSC-3A6 cells) was normalized with the spectrum counts of an internal control, paxillin. Finally, the ratio of protein abundance (normalized non-silencing spectrum counts/normalized GEF-H1-silencing spectrum counts) was calculated to represent the relative abundance of each protein in FAs, and to form the GEF-H1-modulated FA proteome. Bottom inset: western blot of paxillin abundance in FAs isolated from non-silencing and GEF-H1-silencing MSC-3A6 cells treated with Dex (0.1μM, 6h). Fold enrichment (fold) of paxillin in isolated FAs was determined by western blotting (equal total protein loaded). Data are mean±s.e.m (n=3 experiments). (B) Pie diagrams showing the percentage of proteins in six categories. In non-silencing FAs, the reproducibly identified proteins (321 proteins) were categorized: focal adhesion (49 proteins; 15%), cytoskeleton (66 proteins; 21%), extracellular matrix (20 proteins; 6%), plasma membrane (97 proteins; 30%), cytoplasm (77 proteins; 24%), and uncharacterized (12 proteins; 4%). In GEF-H1-silencing FAs, the reproducibly identified proteins (250 proteins) were categorized: focal adhesion (40 proteins; 16%), cytoskeleton (49 proteins; 20%), extracellular matrix (18 proteins; 7%), plasma membrane (69 proteins; 28%), cytoplasm (66 proteins; 26%) or uncharacterized (8 proteins; 3%).

**Fig. S3. Collective modulation by GEF-H1 of the FA-associated abundant proteins in biological pathways.**

Greater than two-fold difference in protein abundance (ratio of protein abundance < 0.5 or > 2) was considered significant. Proteins with white boxes were not detected. The detected proteins were represented by color-coded boxes based on the magnitude of the ratio. The scale was indicated at the bottom. (A) GEF-H1 promotes the recruitment of proteins that serve as positive regulators of actin mechanics, but reduces the recruitment of negative regulators in actin mechanics. (B) GEF-H1 mediates the enrichment of GEF-H1-interacting proteins in FAs. (C) GEF-H1 regulates FA enrichment of proteins in the signaling pathway of RhoA-mediated actomyosin contractility.

**Fig. S4. GEF-H1 mediates the recruitment of NMIIB in FAs under osteogenic induction condition.**

(A) Cell lysate from non-silencing and GEF-H1-silencing MSCs expressing pLKO-vector (mock), pLKO-GEF-H1, or pLKO-GEF-H1_DH(m) were analyzed by western blotting. (B) Cell lysate from non-silencing, NMIIB-silencing, and GEF-H1/NMIIB-silencing MSCs were analyzed by western blotting. (C) TIRF microscopy images of immunolocalized paxillin (red) and NMIIB (green) in non-silencing and GEF-H1-silencing MSCs expressing pLKO-vector (mock), pLKO-GEF-H1, or pLKO-GEF-H1_DH(m) and treated with OIM for 48h. Bar, 20 μm. The 20 μm x 20 μm area indicated in the top images are magnified in the images below. (D) Ratio of average density (intensity per $\mu m^2$) of paxillin or NMIIB within segmented FAs of non-silencing MSCs relative to GEF-H1-silencing MSCs expressing mock (a), GEF-H1-silencing MSCs expressing pLKO-GEF-H1 relative to mock (b) or GEF-H1-silencing MSCs expressing pLKO-GEF-H1_DH(m) relative to mock (c) (n=10 cells for each condition). Data are mean±s.e.m. *$p<0.05$, **$p<0.005$.

**Huang_Supplemental tables**

**Table S1: Categorization of proteins reproducibly identified from non-silencing MSC-3A6 cells.**

| Cellular Component | Gene Name | Entry Name | Common Protein Name |
|---|---|---|---|
| focal adhesion | *ACTB* | ACTB | Actin, cytoplasmic 1 |
| focal adhesion | *ACTN1* | ACTN1 | Alpha-actinin-1 |
| focal adhesion | *CFL1* | COF1 | Cofilin-1 |
| focal adhesion | *CSRP1* | CSRP1 | Cysteine and glycine-rich protein 1 |
| focal adhesion | *CTTN* | SRC8 | Src substrate cortactin |
| focal adhesion | *ENAH* | ENAH | Protein enabled homolog |
| focal adhesion | *EPHA2* | EPHA2 | Ephrin type-A receptor 2 |
| focal adhesion | *EZR* | EZRI | Ezrin |
| focal adhesion | *FBLIM1* | FBLI1 | Filamin-binding LIM protein 1 |
| focal adhesion | *FERMT2* | FERM2 | Fermitin family homolog 2 |
| focal adhesion | *FHL2* | FHL2 | Four and a half LIM domains protein 2 |
| focal adhesion | *FLNA* | FLNA | Filamin-A |
| focal adhesion | *HRAS* | RASH | GTPase HRas |
| focal adhesion | *HSPB1* | HSPB1 | Heat shock protein beta-1 |
| focal adhesion | *ILK* | ILK | Integrin-linked protein kinase |
| focal adhesion | *ITGA2* | ITA2 | Integrin alpha-2 |
| focal adhesion | *ITGA3* | ITA3 | Integrin alpha-3 |
| focal adhesion | *ITGA5* | ITA5 | Integrin alpha-5 |
| focal adhesion | *ITGA6* | ITA6 | Integrin alpha-6 |
| focal adhesion | *ITGA7* | ITA7 | Integrin alpha-7 |
| focal adhesion | *ITGAV* | ITAV | Integrin alpha-V |
| focal adhesion | *ITGB1* | ITB1 | Integrin beta-1 |
| focal adhesion | *ITGB5* | ITB5 | Integrin beta-5 |
| focal adhesion | *LASP1* | LASP1 | LIM and SH3 domain protein 1 |
| focal adhesion | *LIMD1* | LIMD1 | LIM domain-containing protein 1 |
| focal adhesion | *LIMS2* | LIMS2 | LIM and senescent cell antigen-like-containing domain protein 2 |
| focal adhesion | *LPP* | LPP | Lipoma-preferred partner |
| focal adhesion | *LRP1* | LRP1 | Prolow-density lipoprotein receptor-related protein 1 |
| focal adhesion | *MACF1* | MACF1 | Microtubule-actin cross-linking factor 1, isoforms 1/2/3/5 |
| focal adhesion | *MARCKS* | MARCS | Myristoylated alanine-rich C-kinase substrate |
| focal adhesion | *MSN* | MOES | Moesin |
| focal adhesion | *NEXN* | NEXN | Nexilin |

| | | | |
|---|---|---|---|
| focal adhesion | *PALLD* | PALLD | Palladin |
| focal adhesion | *PDLIM2* | PDLI2 | PDZ and LIM domain protein 2 |
| focal adhesion | *PFN1* | PROF1 | Profilin-1 |
| focal adhesion | *PLEC* | PLEC | Plectin |
| focal adhesion | *PRNP* | PRIO | Major prion protein |
| focal adhesion | *PVR* | PVR | Poliovirus receptor |
| focal adhesion | *PXN* | PAXI | Paxillin |
| focal adhesion | *RAC1* | RAC1 | Ras-related C3 botulinum toxin substrate 1 |
| focal adhesion | *TENC1* | TENC1 | Tensin-like C1 domain-containing phosphatase |
| focal adhesion | *TGFB1I1* | TGFI1 | Transforming growth factor beta-1-induced transcript 1 protein |
| focal adhesion | *TLN1* | TLN1 | Talin-1 |
| focal adhesion | *TNS1* | TENS1 | Tensin-1 |
| focal adhesion | *TRIP6* | TRIP6 | Thyroid receptor-interacting protein 6 |
| focal adhesion | *VASP* | VASP | Vasodilator-stimulated phosphoprotein |
| focal adhesion | *VCL* | VINC | Vinculin |
| focal adhesion | *VIM* | VIME | Vimentin |
| focal adhesion | *ZYX* | ZYX | Zyxin |
| cytoskeleton | *ACTN4* | ACTN4 | Alpha-actinin-4 |
| cytoskeleton | *ADD1* | ADDA | Alpha-adducin |
| cytoskeleton | *AGRN* | AGRIN | Agrin |
| cytoskeleton | *AKAP12* | AKA12 | A-kinase anchor protein 12 |
| cytoskeleton | *ALDOA* | ALDOA | Fructose-bisphosphate aldolase A |
| cytoskeleton | *ARHGEF2* | ARHG2 | Rho guanine nucleotide exchange factor 2 |
| cytoskeleton | *ARPC5* | ARPC5 | Actin-related protein 2/3 complex subunit 5 |
| cytoskeleton | *BASP1* | BASP1 | Brain acid soluble protein 1 |
| cytoskeleton | *CALD1* | CALD1 | Caldesmon |
| cytoskeleton | *CALM1* | CALM | Calmodulin |
| cytoskeleton | *CDC42EP3* | BORG2 | Cdc42 effector protein 3 |
| cytoskeleton | *CEP170* | CE170 | Centrosomal protein of 170 kDa |
| cytoskeleton | *CFL2* | COF2 | Cofilin-2 |
| cytoskeleton | *CKAP4* | CKAP4 | Cytoskeleton-associated protein 4 |
| cytoskeleton | *CNN2* | CNN2 | Calponin-2 |
| cytoskeleton | *CNN3* | CNN3 | Calponin-3 |
| cytoskeleton | *CNP* | CN37 | 2',3'-cyclic-nucleotide 3'-phosphodiesterase |
| cytoskeleton | *CORO1C* | COR1C | Coronin-1C |
| cytoskeleton | *CSRP2* | CSRP2 | Cysteine and glycine-rich protein 2 |
| cytoskeleton | *DBN1* | DREB | Drebrin |

| | | | |
|---|---|---|---|
| cytoskeleton | *DBNL* | DBNL | Drebrin-like protein |
| cytoskeleton | *DSTN* | DEST | Destrin |
| cytoskeleton | *FKBP4* | FKBP4 | Peptidyl-prolyl cis-trans isomerase FKBP4 |
| cytoskeleton | *FLNB* | FLNB | Filamin-B |
| cytoskeleton | *FLNC* | FLNC | Filamin-C |
| cytoskeleton | *FLOT1* | FLOT1 | Flotillin-1 |
| cytoskeleton | *GAPDH* | G3P | Glyceraldehyde-3-phosphate dehydrogenase |
| cytoskeleton | *HNRNPA1* | ROA1 | Heterogeneous nuclear ribonucleoprotein A1 |
| cytoskeleton | *INA* | AINX | Alpha-internexin |
| cytoskeleton | *LIMA1* | LIMA1 | LIM domain and actin-binding protein 1 |
| cytoskeleton | *LMNA* | LMNA | Prelamin-A/C |
| cytoskeleton | *MYH10* | MYH10 | Myosin-10 |
| cytoskeleton | *MYH9* | MYH9 | Myosin-9 |
| cytoskeleton | *MYL12A* | ML12A | Myosin regulatory light chain 12A |
| cytoskeleton | *MYL6* | MYL6 | Myosin light polypeptide 6 |
| cytoskeleton | *MYL9* | MYL9 | Myosin regulatory light polypeptide 9 |
| cytoskeleton | *MYO1C* | MYO1C | Unconventional myosin-Ic |
| cytosplasm | *PDIA3* | PDIA3 | Protein disulfide-isomerase A3 |
| cytoskeleton | *PDLIM4* | PDLI4 | PDZ and LIM domain protein 4 |
| cytoskeleton | *PFN2* | PROF2 | Profilin-2 |
| cytoskeleton | *PPP1R12A* | MYPT1 | Protein phosphatase 1 regulatory subunit 12A |
| cytoskeleton | *PPP1R18* | PPR18 | Phostensin |
| cytosplasm | *PRKCSH* | GLU2B | Glucosidase 2 subunit beta |
| cytoskeleton | *RAI14* | RAI14 | Ankycorbin |
| cytosplasm | *RRBP1* | RRBP1 | Ribosome-binding protein 1 |
| cytoskeleton | *S100A11* | S10AB | Protein S100-A11 |
| cytoskeleton | *SEPT2* | SEPT2 | Septin-2 |
| cytoskeleton | *SEPT7* | SEPT7 | Septin-7 |
| cytoskeleton | *SEPT9* | SEPT9 | Septin-9 |
| cytosplasm | *SERPINH1* | SERPH | Serpin H1 |
| cytoskeleton | *SNTB2* | SNTB2 | Beta-2-syntrophin |
| cytoskeleton | *SPTAN1* | SPTN1 | Spectrin alpha chain, non-erythrocytic 1 |
| cytoskeleton | *SPTBN1* | SPTB2 | Spectrin beta chain, non-erythrocytic 1 |
| cytoskeleton | *STMN1* | STMN1 | Stathmin |
| cytoskeleton | *STOM* | STOM | Erythrocyte band 7 integral membrane protein |
| cytoskeleton | *TAGLN2* | TAGL2 | Transgelin-2 |
| cytoskeleton | *TLN2* | TLN2 | Talin-2 |

| | | | |
|---|---|---|---|
| cytoskeleton | *TMOD3* | TMOD3 | Tropomodulin-3 |
| cytoskeleton | *TPM2* | TPM2 | Tropomyosin beta chain |
| cytoskeleton | *TPM3* | TPM3 | Tropomyosin alpha-3 chain |
| cytoskeleton | *TPM4* | TPM4 | Tropomyosin alpha-4 chain |
| cytoskeleton | *TRIOBP* | TARA | TRIO and F-actin-binding protein |
| cytoskeleton | *TUBA1A* | TBA1A | Tubulin alpha-1A chain |
| cytoskeleton | *TUBB2A* | TBB2A | Tubulin beta-2A chain |
| cytoskeleton | *UACA* | UACA | Uveal autoantigen with coiled-coil domains and ankyrin repeats |
| cytoskeleton | *UTRN* | UTRO | Utrophin |
| extracellular matrix | *ADAMTSL1* | ATL1 | ADAMTS-like protein 1 |
| extracellular matrix | *AHSG* | FETUA | Alpha-2-HS-glycoprotein |
| extracellular matrix | *COL1A1* | CO1A1 | Collagen alpha-1(I) chain |
| extracellular matrix | *COL1A2* | CO1A2 | Collagen alpha-2(I) chain |
| extracellular matrix | *COL6A3* | CO6A3 | Collagen alpha-3(VI) chain |
| extracellular matrix | *EMILIN1* | EMIL1 | EMILIN-1 |
| extracellular matrix | *FBLN1* | FBLN1 | Fibulin-1 |
| extracellular matrix | *FBLN2* | FBLN2 | Fibulin-2 |
| extracellular matrix | *FN1* | FINC | Fibronectin |
| extracellular matrix | *HAPLN1* | HPLN1 | Hyaluronan and proteoglycan link protein 1 |
| extracellular matrix | *HAPLN3* | HPLN3 | Hyaluronan and proteoglycan link protein 3 |
| extracellular matrix | *LOX* | LYOX | Protein-lysine 6-oxidase |
| extracellular matrix | *LOXL1* | LOXL1 | Lysyl oxidase homolog 1 |
| extracellular matrix | *LTBP2* | LTBP2 | Latent-transforming growth factor beta-binding protein 2 |
| extracellular matrix | *LTBP4* | LTBP4 | Latent-transforming growth factor beta-binding protein 4 |
| extracellular matrix | *MGP* | MGP | Matrix Gla protein |
| extracellular matrix | *PCOLCE* | PCOC1 | Procollagen C-endopeptidase enhancer 1 |
| extracellular matrix | *TGFB2* | TGFB2 | Transforming growth factor beta-2 |
| extracellular matrix | *VCAN* | CSPG2 | Versican core protein |
| extracellular matrix | *VTN* | VTNC | Vitronectin |
| plasma membrane | *ADAM9* | ADAM9 | Disintegrin and metalloproteinase domain-containing protein 9 |
| plasma membrane | *ADRM1* | ADRM1 | Proteasomal ubiquitin receptor ADRM1 |
| plasma membrane | *ALCAM* | CD166 | CD166 antigen |
| plasma membrane | *ANXA1* | ANXA1 | Annexin A1 |
| plasma membrane | *ANXA2* | ANXA2 | Annexin A2 |
| plasma membrane | *AP2A1* | AP2A1 | AP-2 complex subunit alpha-1 |
| plasma membrane | *AP2M1* | AP2M1 | AP-2 complex subunit mu |
| plasma membrane | *APOC3* | APOC3 | Apolipoprotein C-III |

| | | | |
|---|---|---|---|
| plasma membrane | *APOE* | APOE | Apolipoprotein E |
| plasma membrane | *APOH* | APOH | Beta-2-glycoprotein 1 |
| plasma membrane | *APP* | A4 | Amyloid beta A4 protein |
| plasma membrane | *ATP1A1* | AT1A1 | Sodium/potassium-transporting ATPase subunit alpha-1 |
| plasma membrane | *ATP2B4* | AT2B4 | Plasma membrane calcium-transporting ATPase 4 |
| plasma membrane | *ATP5A1* | ATPA | ATP synthase subunit alpha, mitochondrial |
| plasma membrane | *ATP5O* | ATPO | ATP synthase subunit O, mitochondrial |
| plasma membrane | *BCAM* | BCAM | Basal cell adhesion molecule |
| plasma membrane | *BSG* | BASI | Basigin |
| plasma membrane | *CCDC80* | CCD80 | Coiled-coil domain-containing protein 80 |
| plasma membrane | *CD151* | CD151 | CD151 antigen |
| plasma membrane | *CD44* | CD44 | CD44 antigen |
| plasma membrane | *CD55* | DAF | Complement decay-accelerating factor |
| plasma membrane | *CD59* | CD59 | CD59 glycoprotein |
| plasma membrane | *CD99* | CD99 | CD99 antigen |
| plasma membrane | *CDC42EP1* | BORG5 | Cdc42 effector protein 1 |
| plasma membrane | *CDH2* | CADH2 | Cadherin-2 |
| plasma membrane | *CLIC1* | CLIC1 | Chloride intracellular channel protein 1 |
| plasma membrane | *CLTA* | CLCA | Clathrin light chain A |
| plasma membrane | *CSDA* | DBPA | DNA-binding protein A |
| plasma membrane | *CTGF* | CTGF | Connective tissue growth factor |
| plasma membrane | *CTNNA1* | CTNA1 | Catenin alpha-1 |
| plasma membrane | *CTNNB1* | CTNB1 | Catenin beta-1 |
| plasma membrane | *CTNND1* | CTND1 | Catenin delta-1 |
| plasma membrane | *DKK1* | DKK1 | Dickkopf-related protein 1 |
| plasma membrane | *EDIL3* | EDIL3 | EGF-like repeat and discoidin I-like domain-containing protein 3 |
| plasma membrane | *EEF1A1* | EF1A1 | Elongation factor 1-alpha 1 |
| plasma membrane | *EFEMP2* | FBLN4 | EGF-containing fibulin-like extracellular matrix protein 2 |
| plasma membrane | *EGFR* | EGFR | Epidermal growth factor receptor |
| plasma membrane | *EHD1* | EHD1 | EH domain-containing protein 1 |
| plasma membrane | *EHD4* | EHD4 | EH domain-containing protein 4 |
| plasma membrane | *EPB41L2* | E41L2 | Band 4.1-like protein 2 |
| plasma membrane | *EPHB2* | EPHB2 | Ephrin type-B receptor 2 |
| plasma membrane | *EPS15L1* | EP15R | Epidermal growth factor receptor substrate 15-like 1 |
| plasma membrane | *EPS8* | EPS8 | Epidermal growth factor receptor kinase substrate 8 |
| plasma membrane | *ERBB2IP* | LAP2 | Protein LAP2 |
| plasma membrane | *ESYT1* | ESYT1 | Extended synaptotagmin-1 |

| | | | |
|---|---|---|---|
| plasma membrane | *F5* | FA5 | Coagulation factor V |
| plasma membrane | *GNAI2* | GNAI2 | Guanine nucleotide-binding protein G(i) subunit alpha-2 |
| plasma membrane | *GNAI3* | GNAI3 | Guanine nucleotide-binding protein G(k) subunit alpha |
| plasma membrane | *GNAS* | GNAS1 | Guanine nucleotide-binding protein G(s) subunit alpha isoforms XLas |
| plasma membrane | *GNG12* | GBG12 | Guanine nucleotide-binding protein G(I)/G(S)/G(O) subunit gamma-12 |
| plasma membrane | *GSK3B* | GSK3B | Glycogen synthase kinase-3 beta |
| plasma membrane | *HNRNPM* | HNRPM | Heterogeneous nuclear ribonucleoprotein M |
| plasma membrane | *HSPA5* | GRP78 | 78 kDa glucose-regulated protein |
| plasma membrane | *HSPA8* | HSP7C | Heat shock cognate 71 kDa protein |
| plasma membrane | *HSPA9* | GRP75 | Stress-70 protein, mitochondrial |
| plasma membrane | *HSPD1* | CH60 | 60 kDa heat shock protein, mitochondrial |
| plasma membrane | *HSPG2* | PGBM | Basement membrane-specific heparan sulfate proteoglycan core protein |
| plasma membrane | *ICAM1* | ICAM1 | Intercellular adhesion molecule 1 |
| plasma membrane | *IGF2* | IGF2 | Insulin-like growth factor II |
| plasma membrane | *KIRREL* | KIRR1 | Kin of IRRE-like protein 1 |
| plasma membrane | *LGALS1* | LEG1 | Galectin-1 |
| plasma membrane | *LIN7C* | LIN7C | Protein lin-7 homolog C |
| plasma membrane | *MARCKSL1* | MRP | MARCKS-related protein |
| plasma membrane | *MCAM* | MUC18 | Cell surface glycoprotein MUC18 |
| plasma membrane | *MRC2* | MRC2 | C-type mannose receptor 2 |
| plasma membrane | *MYADM* | MYADM | Myeloid-associated differentiation marker |
| plasma membrane | *MYOF* | MYOF | Myoferlin |
| plasma membrane | *NRP1* | NRP1 | Neuropilin-1 |
| plasma membrane | *NUMB* | NUMB | Protein numb homolog |
| plasma membrane | *PIP4K2A* | PI42A | Phosphatidylinositol 5-phosphate 4-kinase type-2 alpha |
| plasma membrane | *PKM* | KPYM | Pyruvate kinase isozymes M1/M2 |
| plasma membrane | *PLP2* | PLP2 | Proteolipid protein 2 |
| plasma membrane | *PLSCR3* | PLS3 | Phospholipid scramblase 3 |
| plasma membrane | *PLXNB2* | PLXB2 | Plexin-B2 |
| plasma membrane | *PPFIBP1* | LIPB1 | Liprin-beta-1 |
| plasma membrane | *PRSS12* | NETR | Neurotrypsin |
| plasma membrane | *PTK7* | PTK7 | Inactive tyrosine-protein kinase 7 |
| plasma membrane | *RAB13* | RAB13 | Ras-related protein Rab-13 |
| plasma membrane | *RFTN1* | RFTN1 | Raftlin |
| plasma membrane | *RPL7A* | RL7A | 60S ribosomal protein L7a |
| plasma membrane | *SDPR* | SDPR | Serum deprivation-response protein |
| plasma membrane | *SERBP1* | PAIRB | Plasminogen activator inhibitor 1 RNA-binding protein |

| | | | |
|---|---|---|---|
| plasma membrane | *SERPINE1* | PAI1 | Plasminogen activator inhibitor 1 |
| plasma membrane | *SLC1A5* | AAAT | Neutral amino acid transporter B(0) |
| plasma membrane | *SLC39A14* | S39AE | Zinc transporter ZIP14 |
| plasma membrane | *SLC7A5* | LAT1 | Large neutral amino acids transporter small subunit 1 |
| plasma membrane | *SNAP29* | SNP29 | Synaptosomal-associated protein 29 |
| plasma membrane | *STEAP3* | STEA3 | Metalloreductase STEAP3 |
| plasma membrane | *STXBP1* | STXB1 | Syntaxin-binding protein 1 |
| plasma membrane | *TFRC* | TFR1 | Transferrin receptor protein 1 |
| plasma membrane | *TGFB1* | TGFB1 | Transforming growth factor beta-1 |
| plasma membrane | *THBS1* | TSP1 | Thrombospondin-1 |
| plasma membrane | *TJP2* | ZO2 | Tight junction protein ZO-2 |
| plasma membrane | *TNC* | TENA | Tenascin |
| plasma membrane | *VAMP5* | VAMP5 | Vesicle-associated membrane protein 5 |
| plasma membrane | *VASN* | VASN | Vasorin |
| plasma membrane | *WNT5A* | WNT5A | Protein Wnt-5a |
| uncharacterized | *A6NL28* | TPM3L | Putative tropomyosin alpha-3 chain-like protein |
| uncharacterized | *ADAM5P* | ADAM5 | Putative disintegrin and metalloproteinase domain-containing protein 5 |
| uncharacterized | *AKAP2* | AKAP2 | A-kinase anchor protein 2 |
| uncharacterized | *C16orf55* | CP055 | Uncharacterized protein C16orf55 |
| uncharacterized | *CYR61* | CYR61 | Protein CYR61 |
| uncharacterized | *FAM176B* | F176B | Protein FAM176B |
| uncharacterized | *HBA1* | HBA | Hemoglobin subunit alpha |
| uncharacterized | *HIST1H4A* | H4 | Histone H4 |
| uncharacterized | *HPCA* | HPCA | Neuron-specific calcium-binding protein hippocalcin |
| uncharacterized | *LSM12* | LSM12 | Protein LSM12 homolog |
| uncharacterized | *PHLDB1* | PHLB1 | Pleckstrin homology-like domain family B member 1 |
| uncharacterized | *PRKCDBP* | PRDBP | Protein kinase C delta-binding protein |
| cytoplasm | *ADAMTS1* | ATS1 | A disintegrin and metalloproteinase with thrombospondin motifs 1 |
| cytoplasm | *ATP5D* | ATPD | ATP synthase subunit delta, mitochondrial |
| cytoplasm | *ATP5I* | ATP5I | ATP synthase subunit e, mitochondrial |
| cytoplasm | *CCDC50* | CCD50 | Coiled-coil domain-containing protein 50 |
| cytoplasm | *CHCHD3* | CHCH3 | Coiled-coil-helix-coiled-coil-helix domain-containing protein 3, mitochondrial |
| cytoplasm | *CLEC3B* | TETN | Tetranectin |
| cytoplasm | *CLTB* | CLCB | Clathrin light chain B |
| cytoplasm | *CLTC* | CLH1 | Clathrin heavy chain 1 |
| cytoplasm | *COX5A* | COX5A | Cytochrome c oxidase subunit 5A, mitochondrial |
| cytoplasm | *DDX3X* | DDX3X | ATP-dependent RNA helicase DDX3X |

| | | | |
|---|---|---|---|
| cytoplasm | *DPYSL2* | DPYL2 | Dihydropyrimidinase-related protein 2 |
| cytoplasm | *DTD1* | DTD1 | D-tyrosyl-tRNA(Tyr) deacylase 1 |
| cytoplasm | *EEF1D* | EF1D | Elongation factor 1-delta |
| cytoplasm | *EEF2* | EF2 | Elongation factor 2 |
| cytoplasm | *EIF2S1* | IF2A | Eukaryotic translation initiation factor 2 subunit 1 |
| cytoplasm | *EIF4H* | IF4H | Eukaryotic translation initiation factor 4H |
| cytoplasm | *ENSA* | ENSA | Alpha-endosulfine |
| cytoplasm | *EPN2* | EPN2 | Epsin-2 |
| cytoplasm | *FH* | FUMH | Fumarate hydratase, mitochondrial |
| cytoplasm | *GAS6* | GAS6 | Growth arrest-specific protein 6 |
| cytoplasm | *GLIPR2* | GAPR1 | Golgi-associated plant pathogenesis-related protein 1 |
| cytoplasm | *HNRNPA2B1* | ROA2 | Heterogeneous nuclear ribonucleoproteins A2/B1 |
| cytoplasm | *HNRNPD* | HNRPD | Heterogeneous nuclear ribonucleoprotein D0 |
| cytoplasm | *HTRA1* | HTRA1 | Serine protease HTRA1 |
| cytoplasm | *KANK2* | KANK2 | KN motif and ankyrin repeat domain-containing protein 2 |
| cytoplasm | *KPNB1* | IMB1 | Importin subunit beta-1 |
| cytoplasm | *LMO7* | LMO7 | LIM domain only protein 7 |
| cytoplasm | *MIF* | MIF | Macrophage migration inhibitory factor |
| cytoplasm | *MT1E* | MT1E | Metallothionein-1E |
| cytoplasm | *MT2A* | MT2 | Metallothionein-2 |
| cytoplasm | *NACA* | NACA | Nascent polypeptide-associated complex subunit alpha |
| cytoplasm | *NDUFA2* | NDUA2 | NADH dehydrogenase [ubiquinone] 1 alpha subcomplex subunit 2 |
| cytoplasm | *NPM1* | NPM | Nucleophosmin |
| cytoplasm | *PCBP1* | PCBP1 | Poly(rC)-binding protein 1 |
| cytoplasm | *PCMT1* | PIMT | Protein-L-isoaspartate(D-aspartate) O-methyltransferase |
| cytoplasm | *PGAM1* | PGAM1 | Phosphoglycerate mutase 1 |
| cytoplasm | *PPIA* | PPIA | Peptidyl-prolyl cis-trans isomerase A |
| cytoplasm | *PPIB* | PPIB | Peptidyl-prolyl cis-trans isomerase B |
| cytoplasm | *PRDX1* | PRDX1 | Peroxiredoxin-1 |
| cytoplasm | *PTRF* | PTRF | Polymerase I and transcript release factor |
| cytoplasm | *RAB21* | RAB21 | Ras-related protein Rab-21 |
| cytoplasm | *RPL11* | RL11 | 60S ribosomal protein L11 |
| cytoplasm | *RPL12* | RL12 | 60S ribosomal protein L12 |
| cytoplasm | *RPL13* | RL13 | 60S ribosomal protein L13 |
| cytoplasm | *RPL22* | RL22 | 60S ribosomal protein L22 |
| cytoplasm | *RPL23A* | RL23A | 60S ribosomal protein L23a |
| cytoplasm | *RPL29* | RL29 | 60S ribosomal protein L29 |

| | | | |
|---|---|---|---|
| cytoplasm | *RPL30* | RL30 | 60S ribosomal protein L30 |
| cytoplasm | *RPL35A* | RL35A | 60S ribosomal protein L35a |
| cytoplasm | *RPL5* | RL5 | 60S ribosomal protein L5 |
| cytoplasm | *RPL6* | RL6 | 60S ribosomal protein L6 |
| cytoplasm | *RPLP1* | RLA1 | 60S acidic ribosomal protein P1 |
| cytoplasm | *RPS18* | RS18 | 40S ribosomal protein S18 |
| cytoplasm | *RPS19* | RS19 | 40S ribosomal protein S19 |
| cytoplasm | *RPS2* | RS2 | 40S ribosomal protein S2 |
| cytoplasm | *RPS21* | RS21 | 40S ribosomal protein S21 |
| cytoplasm | *RPS25* | RS25 | 40S ribosomal protein S25 |
| cytoplasm | *RPS28* | RS28 | 40S ribosomal protein S28 |
| cytoplasm | *RPS29* | RS29 | 40S ribosomal protein S29 |
| cytoplasm | *RPS3A* | RS3A | 40S ribosomal protein S3a |
| cytoplasm | *RPS6* | RS6 | 40S ribosomal protein S6 |
| cytoplasm | *SCAMP3* | SCAM3 | Secretory carrier-associated membrane protein 3 |
| cytoplasm | *SEPT8* | SEPT8 | Septin-8 |
| cytoplasm | *SH3BGRL3* | SH3L3 | SH3 domain-binding glutamic acid-rich-like protein 3 |
| cytoplasm | *SRP14* | SRP14 | Signal recognition particle 14 kDa protein |
| cytoplasm | *SRP9* | SRP09 | Signal recognition particle 9 kDa protein |
| cytoplasm | *STX7* | STX7 | Syntaxin-7 |
| cytoplasm | *THSD4* | THSD4 | Thrombospondin type-1 domain-containing protein 4 |
| cytoplasm | *TPI1* | TPIS | Triosephosphate isomerase |
| cytoplasm | *TUFM* | EFTU | Elongation factor Tu, mitochondrial |
| cytoplasm | *UQCRC1* | QCR1 | Cytochrome b-c1 complex subunit 1, mitochondrial |
| cytoplasm | *VAT1* | VAT1 | Synaptic vesicle membrane protein VAT-1 homolog |
| cytoplasm | *VCP* | TERA | Transitional endoplasmic reticulum ATPase |
| cytoplasm | *YBX1* | YBOX1 | Nuclease-sensitive element-binding protein 1 |
| cytoplasm | *YWHAQ* | 1433T | 14-3-3 protein theta |
| cytoplasm | *YWHAZ* | 1433Z | 14-3-3 protein zeta/delta |
| cytoplasm | *ZC3H15* | ZC3HF | Zinc finger CCCH domain-containing protein 15 |

**Table S2: Categorization of proteins reproducibly identified from GEF-H1-silencing MSC-3A6 cells.**

| Cellular Component | Gene Name | Entry Name | Common Protein Name |
|---|---|---|---|
| focal adhesion | *ACTB* | ACTB | Actin, cytoplasmic 1 |
| focal adhesion | *ACTN1* | ACTN1 | Alpha-actinin-1 |
| focal adhesion | *CFL1* | COF1 | Cofilin-1 |
| focal adhesion | *CSRP1* | CSRP1 | Cysteine and glycine-rich protein 1 |
| focal adhesion | *CTTN* | SRC8 | Src substrate cortactin |
| focal adhesion | *ENAH* | ENAH | Protein enabled homolog |
| focal adhesion | *EPHA2* | EPHA2 | Ephrin type-A receptor 2 |
| focal adhesion | *EZR* | EZRI | Ezrin |
| focal adhesion | *FHL2* | FHL2 | Four and a half LIM domains protein 2 |
| focal adhesion | *FLNA* | FLNA | Filamin-A |
| focal adhesion | *HRAS* | RASH | GTPase HRas |
| focal adhesion | *HSPB1* | HSPB1 | Heat shock protein beta-1 |
| focal adhesion | *ILK* | ILK | Integrin-linked protein kinase |
| focal adhesion | *ITGA2* | ITA2 | Integrin alpha-2 |
| focal adhesion | *ITGA3* | ITA3 | Integrin alpha-3 |
| focal adhesion | *ITGA5* | ITA5 | Integrin alpha-5 |
| focal adhesion | *ITGA6* | ITA6 | Integrin alpha-6 |
| focal adhesion | *ITGAV* | ITAV | Integrin alpha-V |
| focal adhesion | *ITGB1* | ITB1 | Integrin beta-1 |
| focal adhesion | *ITGB5* | ITB5 | Integrin beta-5 |
| focal adhesion | *LASP1* | LASP1 | LIM and SH3 domain protein 1 |
| focal adhesion | *LIMD1* | LIMD1 | LIM domain-containing protein 1 |
| focal adhesion | *LIMS2* | LIMS2 | LIM and senescent cell antigen-like-containing domain protein 2 |
| focal adhesion | *LRP1* | LRP1 | Prolow-density lipoprotein receptor-related protein 1 |
| focal adhesion | *MARCKS* | MARCS | Myristoylated alanine-rich C-kinase substrate |
| focal adhesion | *MSN* | MOES | Moesin |
| focal adhesion | *NEXN* | NEXN | Nexilin |
| focal adhesion | *PALLD* | PALLD | Palladin |
| focal adhesion | *PDLIM2* | PDLI2 | PDZ and LIM domain protein 2 |
| focal adhesion | *PFN1* | PROF1 | Profilin-1 |
| focal adhesion | *PLEC* | PLEC | Plectin |
| focal adhesion | *PRNP* | PRIO | Major prion protein |
| focal adhesion | *PXN* | PAXI | Paxillin |
| focal adhesion | *TENC1* | TENC1 | Tensin-like C1 domain-containing phosphatase |

| | | | |
|---|---|---|---|
| focal adhesion | *TLN1* | TLN1 | Talin-1 |
| focal adhesion | *TNS1* | TENS1 | Tensin-1 |
| focal adhesion | *VASP* | VASP | Vasodilator-stimulated phosphoprotein |
| focal adhesion | *VCL* | VINC | Vinculin |
| focal adhesion | *VIM* | VIME | Vimentin |
| focal adhesion | *ZYX* | ZYX | Zyxin |
| cytoskeleton | *ACTN4* | ACTN4 | Alpha-actinin-4 |
| cytoskeleton | *ADD1* | ADDA | Alpha-adducin |
| cytoskeleton | *AGRN* | AGRIN | Agrin |
| cytoskeleton | *AKAP12* | AKA12 | A-kinase anchor protein 12 |
| cytoskeleton | *ALDOA* | ALDOA | Fructose-bisphosphate aldolase A |
| cytoskeleton | *BASP1* | BASP1 | Brain acid soluble protein 1 |
| cytoskeleton | *CALD1* | CALD1 | Caldesmon |
| cytoskeleton | *CALM1* | CALM | Calmodulin |
| cytoskeleton | *CEP170* | CE170 | Centrosomal protein of 170 kDa |
| cytoskeleton | *CFL2* | COF2 | Cofilin-2 |
| cytoskeleton | *CNN2* | CNN2 | Calponin-2 |
| cytoskeleton | *CNN3* | CNN3 | Calponin-3 |
| cytoskeleton | *CORO1C* | COR1C | Coronin-1C |
| cytoskeleton | *CSRP2* | CSRP2 | Cysteine and glycine-rich protein 2 |
| cytoskeleton | *DBN1* | DREB | Drebrin |
| cytoskeleton | *DBNL* | DBNL | Drebrin-like protein |
| cytoskeleton | *DSTN* | DEST | Destrin |
| cytoskeleton | *FKBP4* | FKBP4 | Peptidyl-prolyl cis-trans isomerase FKBP4 |
| cytoskeleton | *FLNB* | FLNB | Filamin-B |
| cytoskeleton | *FLNC* | FLNC | Filamin-C |
| cytoskeleton | *FLOT1* | FLOT1 | Flotillin-1 |
| cytoskeleton | *GAPDH* | G3P | Glyceraldehyde-3-phosphate dehydrogenase |
| cytoskeleton | *INA* | AINX | Alpha-internexin |
| cytoskeleton | *LIMA1* | LIMA1 | LIM domain and actin-binding protein 1 |
| cytoskeleton | *LMNA* | LMNA | Prelamin-A/C |
| cytoskeleton | *MYH9* | MYH9 | Myosin-9 |
| cytoskeleton | *MYL12A* | ML12A | Myosin regulatory light chain 12A |
| cytoskeleton | *MYL6* | MYL6 | Myosin light polypeptide 6 |
| cytoskeleton | *MYO1C* | MYO1C | Unconventional myosin-Ic |
| cytosplasm | *PDIA3* | PDIA3 | Protein disulfide-isomerase A3 |
| cytoskeleton | *PPP1R18* | PPR18 | Phostensin |

| | | | |
|---|---|---|---|
| cytosplasm | *PRKCSH* | GLU2B | Glucosidase 2 subunit beta |
| cytosplasm | *RRBP1* | RRBP1 | Ribosome-binding protein 1 |
| cytoskeleton | *SEPT2* | SEPT2 | Septin-2 |
| cytoskeleton | *SEPT7* | SEPT7 | Septin-7 |
| cytoskeleton | *SEPT9* | SEPT9 | Septin-9 |
| cytosplasm | *SERPINH1* | SERPH | Serpin H1 |
| cytoskeleton | *SPTAN1* | SPTN1 | Spectrin alpha chain, non-erythrocytic 1 |
| cytoskeleton | *SPTBN1* | SPTB2 | Spectrin beta chain, non-erythrocytic 1 |
| cytoskeleton | *STMN1* | STMN1 | Stathmin |
| cytoskeleton | *STOM* | STOM | Erythrocyte band 7 integral membrane protein |
| cytoskeleton | *TAGLN2* | TAGL2 | Transgelin-2 |
| cytoskeleton | *TPM2* | TPM2 | Tropomyosin beta chain |
| cytoskeleton | *TPM3* | TPM3 | Tropomyosin alpha-3 chain |
| cytoskeleton | *TPM4* | TPM4 | Tropomyosin alpha-4 chain |
| cytoskeleton | *TRIOBP* | TARA | TRIO and F-actin-binding protein |
| cytoskeleton | *TUBA1A* | TBA1A | Tubulin alpha-1A chain |
| cytoskeleton | *TUBB2A* | TBB2A | Tubulin beta-2A chain |
| cytoskeleton | *UTRN* | UTRO | Utrophin |
| extracellular matrix | *ADAMTSL1* | ATL1 | ADAMTS-like protein 1 |
| extracellular matrix | *AHSG* | FETUA | Alpha-2-HS-glycoprotein |
| extracellular matrix | *COL1A1* | CO1A1 | Collagen alpha-1(I) chain |
| extracellular matrix | *COL1A2* | CO1A2 | Collagen alpha-2(I) chain |
| extracellular matrix | *COL6A3* | CO6A3 | Collagen alpha-3(VI) chain |
| extracellular matrix | *EMILIN1* | EMIL1 | EMILIN-1 |
| extracellular matrix | *FBLN1* | FBLN1 | Fibulin-1 |
| extracellular matrix | *FBLN2* | FBLN2 | Fibulin-2 |
| extracellular matrix | *FN1* | FINC | Fibronectin |
| extracellular matrix | *HAPLN3* | HPLN3 | Hyaluronan and proteoglycan link protein 3 |
| extracellular matrix | *LOX* | LYOX | Protein-lysine 6-oxidase |
| extracellular matrix | *LOXL1* | LOXL1 | Lysyl oxidase homolog 1 |
| extracellular matrix | *LTBP2* | LTBP2 | Latent-transforming growth factor beta-binding protein 2 |
| extracellular matrix | *LTBP4* | LTBP4 | Latent-transforming growth factor beta-binding protein 4 |
| extracellular matrix | *MGP* | MGP | Matrix Gla protein |
| extracellular matrix | *PCOLCE* | PCOC1 | Procollagen C-endopeptidase enhancer 1 |
| extracellular matrix | *VCAN* | CSPG2 | Versican core protein |
| extracellular matrix | *VTN* | VTNC | Vitronectin |
| plasma membrane | *ADAM9* | ADAM9 | Disintegrin and metalloproteinase domain-containing protein 9 |

| | | | |
|---|---|---|---|
| plasma membrane | *ALCAM* | CD166 | CD166 antigen |
| plasma membrane | *ANXA1* | ANXA1 | Annexin A1 |
| plasma membrane | *ANXA2* | ANXA2 | Annexin A2 |
| plasma membrane | *AP2A1* | AP2A1 | AP-2 complex subunit alpha-1 |
| plasma membrane | *AP2M1* | AP2M1 | AP-2 complex subunit mu |
| plasma membrane | *APOC3* | APOC3 | Apolipoprotein C-III |
| plasma membrane | *APOE* | APOE | Apolipoprotein E |
| plasma membrane | *APP* | A4 | Amyloid beta A4 protein |
| plasma membrane | *ATP1A1* | AT1A1 | Sodium/potassium-transporting ATPase subunit alpha-1 |
| plasma membrane | *ATP2B4* | AT2B4 | Plasma membrane calcium-transporting ATPase 4 |
| plasma membrane | *ATP5A1* | ATPA | ATP synthase subunit alpha, mitochondrial |
| plasma membrane | *BSG* | BASI | Basigin |
| plasma membrane | *CCDC80* | CCD80 | Coiled-coil domain-containing protein 80 |
| plasma membrane | *CD44* | CD44 | CD44 antigen |
| plasma membrane | *CDH2* | CADH2 | Cadherin-2 |
| plasma membrane | *CLIC1* | CLIC1 | Chloride intracellular channel protein 1 |
| plasma membrane | *CLTA* | CLCA | Clathrin light chain A |
| plasma membrane | *CSDA* | DBPA | DNA-binding protein A |
| plasma membrane | *CTNNA1* | CTNA1 | Catenin alpha-1 |
| plasma membrane | *CTNNB1* | CTNB1 | Catenin beta-1 |
| plasma membrane | *DKK1* | DKK1 | Dickkopf-related protein 1 |
| plasma membrane | *EDIL3* | EDIL3 | EGF-like repeat and discoidin I-like domain-containing protein 3 |
| plasma membrane | *EEF1A1* | EF1A1 | Elongation factor 1-alpha 1 |
| plasma membrane | *EFEMP2* | FBLN4 | EGF-containing fibulin-like extracellular matrix protein 2 |
| plasma membrane | *ENO1* | ENOA | Alpha-enolase |
| plasma membrane | *EPB41L2* | E41L2 | Band 4.1-like protein 2 |
| plasma membrane | *EPHB2* | EPHB2 | Ephrin type-B receptor 2 |
| plasma membrane | *EPS8* | EPS8 | Epidermal growth factor receptor kinase substrate 8 |
| plasma membrane | *ESYT1* | ESYT1 | Extended synaptotagmin-1 |
| plasma membrane | *F2* | THRB | Prothrombin |
| plasma membrane | *F5* | FA5 | Coagulation factor V |
| plasma membrane | *GNAI2* | GNAI2 | Guanine nucleotide-binding protein G(i) subunit alpha-2 |
| plasma membrane | *GNAI3* | GNAI3 | Guanine nucleotide-binding protein G(k) subunit alpha |
| plasma membrane | *GNG12* | GBG12 | Guanine nucleotide-binding protein G(I)/G(S)/G(O) subunit gamma-12 |
| plasma membrane | *GSK3B* | GSK3B | Glycogen synthase kinase-3 beta |
| plasma membrane | *HNRNPM* | HNRPM | Heterogeneous nuclear ribonucleoprotein M |
| plasma membrane | *HSPA5* | GRP78 | 78 kDa glucose-regulated protein |

| | | | |
|---|---|---|---|
| plasma membrane | *HSPA8* | HSP7C | Heat shock cognate 71 kDa protein |
| plasma membrane | *HSPA9* | GRP75 | Stress-70 protein, mitochondrial |
| plasma membrane | *HSPD1* | CH60 | 60 kDa heat shock protein, mitochondrial |
| plasma membrane | *HSPG2* | PGBM | Basement membrane-specific heparan sulfate proteoglycan core protein |
| plasma membrane | *ICAM1* | ICAM1 | Intercellular adhesion molecule 1 |
| plasma membrane | *IGF2* | IGF2 | Insulin-like growth factor II |
| plasma membrane | *LGALS1* | LEG1 | Galectin-1 |
| plasma membrane | *LIN7C* | LIN7C | Protein lin-7 homolog C |
| plasma membrane | *MARCKSL1* | MRP | MARCKS-related protein |
| plasma membrane | *MCAM* | MUC18 | Cell surface glycoprotein MUC18 |
| plasma membrane | *MDH2* | MDHM | Malate dehydrogenase, mitochondrial |
| plasma membrane | *MYADM* | MYADM | Myeloid-associated differentiation marker |
| plasma membrane | *MYOF* | MYOF | Myoferlin |
| plasma membrane | *NRP1* | NRP1 | Neuropilin-1 |
| plasma membrane | *PACSIN3* | PACN3 | Protein kinase C and casein kinase substrate in neurons protein 3 |
| plasma membrane | *PIP4K2A* | PI42A | Phosphatidylinositol 5-phosphate 4-kinase type-2 alpha |
| plasma membrane | *PKM* | KPYM | Pyruvate kinase isozymes M1/M2 |
| plasma membrane | *PPFIBP1* | LIPB1 | Liprin-beta-1 |
| plasma membrane | *PTK7* | PTK7 | Inactive tyrosine-protein kinase 7 |
| plasma membrane | *RFTN1* | RFTN1 | Raftlin |
| plasma membrane | *RPL7A* | RL7A | 60S ribosomal protein L7a |
| plasma membrane | *SDPR* | SDPR | Serum deprivation-response protein |
| plasma membrane | *SERPINE1* | PAI1 | Plasminogen activator inhibitor 1 |
| plasma membrane | *SLC1A5* | AAAT | Neutral amino acid transporter B(0) |
| plasma membrane | *STXBP1* | STXB1 | Syntaxin-binding protein 1 |
| plasma membrane | *TGFB1* | TGFB1 | Transforming growth factor beta-1 |
| plasma membrane | *THBS1* | TSP1 | Thrombospondin-1 |
| plasma membrane | *TJP2* | ZO2 | Tight junction protein ZO-2 |
| plasma membrane | *TNC* | TENA | Tenascin |
| plasma membrane | *VASN* | VASN | Vasorin |
| plasma membrane | *WNT5A* | WNT5A | Protein Wnt-5a |
| uncharacterized | *A6NL28* | TPM3L | Putative tropomyosin alpha-3 chain-like protein |
| uncharacterized | *AKAP2* | AKAP2 | A-kinase anchor protein 2 |
| uncharacterized | *CYR61* | CYR61 | Protein CYR61 |
| uncharacterized | *FAM176B* | F176B | Protein FAM176B |
| uncharacterized | *HBA1* | HBA | Hemoglobin subunit alpha |
| uncharacterized | *HIST1H4A* | H4 | Histone H4 |

| | | | |
|---|---|---|---|
| uncharacterized | *HPCA* | HPCA | Neuron-specific calcium-binding protein hippocalcin |
| uncharacterized | *PRKCDBP* | PRDBP | Protein kinase C delta-binding protein |
| cytoplasm | *ADAMTS1* | ATS1 | A disintegrin and metalloproteinase with thrombospondin motifs 1 |
| cytoplasm | *CCDC50* | CCD50 | Coiled-coil domain-containing protein 50 |
| cytoplasm | *CHCHD3* | CHCH3 | Coiled-coil-helix-coiled-coil-helix domain-containing protein 3, mitochondrial |
| cytoplasm | *CLTB* | CLCB | Clathrin light chain B |
| cytoplasm | *COX5A* | COX5A | Cytochrome c oxidase subunit 5A, mitochondrial |
| cytoplasm | *DPYSL2* | DPYL2 | Dihydropyrimidinase-related protein 2 |
| cytoplasm | *DTD1* | DTD1 | D-tyrosyl-tRNA(Tyr) deacylase 1 |
| cytoplasm | *EEF1B2* | EF1B | Elongation factor 1-beta |
| cytoplasm | *EEF1D* | EF1D | Elongation factor 1-delta |
| cytoplasm | *EEF2* | EF2 | Elongation factor 2 |
| cytoplasm | *EIF2S1* | IF2A | Eukaryotic translation initiation factor 2 subunit 1 |
| cytoplasm | *EIF4H* | IF4H | Eukaryotic translation initiation factor 4H |
| cytoplasm | *ENSA* | ENSA | Alpha-endosulfine |
| cytoplasm | *EPN2* | EPN2 | Epsin-2 |
| cytoplasm | *FH* | FUMH | Fumarate hydratase, mitochondrial |
| cytoplasm | *GLIPR2* | GAPR1 | Golgi-associated plant pathogenesis-related protein 1 |
| cytoplasm | *HTRA1* | HTRA1 | Serine protease HTRA1 |
| cytoplasm | *KANK2* | KANK2 | KN motif and ankyrin repeat domain-containing protein 2 |
| cytoplasm | *LMO7* | LMO7 | LIM domain only protein 7 |
| cytoplasm | *LSM14A* | LS14A | Protein LSM14 homolog A |
| cytoplasm | *MIF* | MIF | Macrophage migration inhibitory factor |
| cytoplasm | *MT1E* | MT1E | Metallothionein-1E |
| cytoplasm | *NACA* | NACA | Nascent polypeptide-associated complex subunit alpha |
| cytoplasm | *NDUFA2* | NDUA2 | NADH dehydrogenase [ubiquinone] 1 alpha subcomplex subunit 2 |
| cytoplasm | *NPM1* | NPM | Nucleophosmin |
| cytoplasm | *PCBP1* | PCBP1 | Poly(rC)-binding protein 1 |
| cytoplasm | *PCMT1* | PIMT | Protein-L-isoaspartate(D-aspartate) O-methyltransferase |
| cytoplasm | *PGAM1* | PGAM1 | Phosphoglycerate mutase 1 |
| cytoplasm | *PLS3* | PLST | Plastin-3 |
| cytoplasm | *PPIA* | PPIA | Peptidyl-prolyl cis-trans isomerase A |
| cytoplasm | *PPP1R12C* | PP12C | Protein phosphatase 1 regulatory subunit 12C |
| cytoplasm | *PTRF* | PTRF | Polymerase I and transcript release factor |
| cytoplasm | *RPL11* | RL11 | 60S ribosomal protein L11 |
| cytoplasm | *RPL12* | RL12 | 60S ribosomal protein L12 |
| cytoplasm | *RPL13* | RL13 | 60S ribosomal protein L13 |

| | | | |
|---|---|---|---|
| cytoplasm | *RPL22* | RL22 | 60S ribosomal protein L22 |
| cytoplasm | *RPL35A* | RL35A | 60S ribosomal protein L35a |
| cytoplasm | *RPL37A* | RL37A | 60S ribosomal protein L37a |
| cytoplasm | *RPL5* | RL5 | 60S ribosomal protein L5 |
| cytoplasm | *RPL9* | RL9 | 60S ribosomal protein L9 |
| cytoplasm | *RPLP1* | RLA1 | 60S acidic ribosomal protein P1 |
| cytoplasm | *RPS18* | RS18 | 40S ribosomal protein S18 |
| cytoplasm | *RPS19* | RS19 | 40S ribosomal protein S19 |
| cytoplasm | *RPS2* | RS2 | 40S ribosomal protein S2 |
| cytoplasm | *RPS21* | RS21 | 40S ribosomal protein S21 |
| cytoplasm | *RPS25* | RS25 | 40S ribosomal protein S25 |
| cytoplasm | *RPS29* | RS29 | 40S ribosomal protein S29 |
| cytoplasm | *RPS3A* | RS3A | 40S ribosomal protein S3a |
| cytoplasm | *RPS6* | RS6 | 40S ribosomal protein S6 |
| cytoplasm | *RPS8* | RS8 | 40S ribosomal protein S8 |
| cytoplasm | *SCAMP3* | SCAM3 | Secretory carrier-associated membrane protein 3 |
| cytoplasm | *SEC22B* | SC22B | Vesicle-trafficking protein SEC22b |
| cytoplasm | *SEPT8* | SEPT8 | Septin-8 |
| cytoplasm | *SH3BGRL3* | SH3L3 | SH3 domain-binding glutamic acid-rich-like protein 3 |
| cytoplasm | *SNRPE* | RUXE | Small nuclear ribonucleoprotein E |
| cytoplasm | *SRP14* | SRP14 | Signal recognition particle 14 kDa protein |
| cytoplasm | *SRP9* | SRP09 | Signal recognition particle 9 kDa protein |
| cytoplasm | *SSBP1* | SSBP | Single-stranded DNA-binding protein, mitochondrial |
| cytoplasm | *THSD4* | THSD4 | Thrombospondin type-1 domain-containing protein 4 |
| cytoplasm | *TPI1* | TPIS | Triosephosphate isomerase |
| cytoplasm | *VAT1* | VAT1 | Synaptic vesicle membrane protein VAT-1 homolog |
| cytoplasm | *VCP* | TERA | Transitional endoplasmic reticulum ATPase |
| cytoplasm | *YBX1* | YBOX1 | Nuclease-sensitive element-binding protein 1 |
| cytoplasm | *YWHAQ* | 1433T | 14-3-3 protein theta |
| cytoplasm | *YWHAZ* | 1433Z | 14-3-3 protein zeta/delta |
| cytoplasm | *ZC3H15* | ZC3HF | Zinc finger CCCH domain-containing protein 15 |

**Table S3: Proteins in the reproducible lists from both non-silencing and GEF-H1-silencing MSC-3A6 cells and their ratios indicating change in relative FA abundance in response to GEF-H1 expression.**

**Only present in GEF-H1-silencing FAs**

| Gene Name | Entry Name | Common Protein Name |
|---|---|---|
| *EEF1B2* | EF1B | Elongation factor 1-beta |
| *ENO1* | ENOA | Alpha-enolase |
| *F2* | THRB | Prothrombin |
| *LSM14A* | LS14A | Protein LSM14 homolog A |
| *MDH2* | MDHM | Malate dehydrogenase, mitochondrial |
| *PACSIN3* | PACN3 | Protein kinase C and casein kinase substrate in neurons protein 3 |
| *PLS3* | PLST | Plastin-3 |
| *PPP1R12C* | PP12C | Protein phosphatase 1 regulatory subunit 12C |
| *RPL37A* | RL37A | 60S ribosomal protein L37a |
| *RPL9* | RL9 | 60S ribosomal protein L9 |
| *RPS8* | RS8 | 40S ribosomal protein S8 |
| *SEC22B* | SC22B | Vesicle-trafficking protein SEC22b |
| *SNRPE* | RUXE | Small nuclear ribonucleoprotein E |
| *SSBP1* | SSBP | Single-stranded DNA-binding protein, mitochondrial |

**FA recruitment inhibited by GEF-H1expression (the Ratio of protein abundance < 0.5)**

| Gene Name | Entry Name | Common Protein Name | Ratio of Protein Abundance |
|---|---|---|---|
| *ADAMTSL1* | ATL1 | ADAMTS-like protein 1 | 0.144844491 |
| *AKAP12* | AKA12 | A-kinase anchor protein 12 | 0.424364482 |
| *AKAP2* | AKAP2 | A-kinase anchor protein 2 | 0.195876861 |
| *ALCAM* | CD166 | CD166 antigen | 0.203510266 |
| *ALDOA* | ALDOA | Fructose-bisphosphate aldolase A | 0.115771842 |
| *ANXA1* | ANXA1 | Annexin A1 | 0.126802293 |
| *ATP1A1* | AT1A1 | Sodium/potassium-transporting ATPase subunit alpha-1 | 0.256730516 |
| *ATP5A1* | ATPA | ATP synthase subunit alpha, mitochondrial | 0.072876591 |
| *BSG* | BASI | Basigin | 0.181119399 |
| *CALD1* | CALD1 | Caldesmon | 0.37990232 |
| *CALM1* | CALM | Calmodulin | 0.296527863 |
| *CD44* | CD44 | CD44 antigen | 0.325641531 |
| *CDH2* | CADH2 | Cadherin-2 | 0.496416112 |

| | | | |
|---|---|---|---|
| *CEP170* | CE170 | Centrosomal protein of 170 kDa | 0.388971872 |
| *CFL1* | COF1 | Cofilin-1 | 0.179149181 |
| *CFL2* | COF2 | Cofilin-2 | 0.073638429 |
| *CLIC1* | CLIC1 | Chloride intracellular channel protein 1 | 0.49882972 |
| *CNN2* | CNN2 | Calponin-2 | 0.190277508 |
| *COL1A1* | CO1A1 | Collagen alpha-1(I) chain | 0.259951104 |
| *COL1A2* | CO1A2 | Collagen alpha-2(I) chain | 0.440716288 |
| *COL6A3* | CO6A3 | Collagen alpha-3(VI) chain | 0.063489858 |
| *CORO1C* | COR1C | Coronin-1C | 0.37030344 |
| *CSDA* | DBPA | DNA-binding protein A | 0.056056181 |
| *DPYSL2* | DPYL2 | Dihydropyrimidinase-related protein 2 | 0.21311785 |
| *DSTN* | DEST | Destrin | 0.126342136 |
| *DTD1* | DTD1 | D-tyrosyl-tRNA(Tyr) deacylase 1 | 0.288355069 |
| *EDIL3* | EDIL3 | EGF-like repeat and discoidin I-like domain-containing protein 3 | 0.245640847 |
| *EPS8* | EPS8 | Epidermal growth factor receptor kinase substrate 8 | 0.045045711 |
| *ESYT1* | ESYT1 | Extended synaptotagmin-1 | 0.213128217 |
| *F5* | FA5 | Coagulation factor V | 0.258574403 |
| *FH* | FUMH | Fumarate hydratase, mitochondrial | 0.206589759 |
| *FHL2* | FHL2 | Four and a half LIM domains protein 2 | 0.420729598 |
| *FKBP4* | FKBP4 | Peptidyl-prolyl cis-trans isomerase FKBP4 | 0.346569252 |
| *FN1* | FINC | Fibronectin | 0.22304462 |
| *GSK3B* | GSK3B | Glycogen synthase kinase-3 beta | 0.427989077 |
| *HIST1H4A* | H4 | Histone H4 | 0.274960501 |
| *HNRNPM* | HNRPM | Heterogeneous nuclear ribonucleoprotein M | 0.417420614 |
| *HSPA5* | GRP78 | 78 kDa glucose-regulated protein | 0.095071193 |
| *HSPB1* | HSPB1 | Heat shock protein beta-1 | 0.121116184 |
| *HSPD1* | CH60 | 60 kDa heat shock protein, mitochondrial | 0.188968036 |
| *HSPG2* | PGBM | Basement membrane-specific heparan sulfate proteoglycan core protein | 0.476719138 |
| *HTRA1* | HTRA1 | Serine protease HTRA1 | 0.248609894 |
| *ICAM1* | ICAM1 | Intercellular adhesion molecule 1 | 0.329409173 |
| *ILK* | ILK | Integrin-linked protein kinase | 0.345276588 |
| *ITGA5* | ITA5 | Integrin alpha-5 | 0.272516581 |
| *ITGAV* | ITAV | Integrin alpha-V | 0.462728948 |
| *LOXL1* | LOXL1 | Lysyl oxidase homolog 1 | 0.187504402 |
| *LTBP4* | LTBP4 | Latent-transforming growth factor beta-binding protein 4 | 0.054551129 |
| *MARCKSL1* | MRP | MARCKS-related protein | 0.455690468 |
| *MGP* | MGP | Matrix Gla protein | 0.361021978 |

| | | | |
|---|---|---|---|
| *MIF* | MIF | Macrophage migration inhibitory factor | 0.169973714 |
| *MYADM* | MYADM | Myeloid-associated differentiation marker | 0.13253645 |
| *MYL12A* | ML12A | Myosin regulatory light chain 12A | 0.412838065 |
| *PALLD* | PALLD | Palladin | 0.285711118 |
| *PCBP1* | PCBP1 | Poly(rC)-binding protein 1 | 0.150489837 |
| *PCMT1* | PIMT | Protein-L-isoaspartate(D-aspartate) O-methyltransferase | 0.061639774 |
| *PCOLCE* | PCOC1 | Procollagen C-endopeptidase enhancer 1 | 0.103570967 |
| *PFN1* | PROF1 | Profilin-1 | 0.148795011 |
| *PPIA* | PPIA | Peptidyl-prolyl cis-trans isomerase A | 0.30787814 |
| *PRNP* | PRIO | Major prion protein | 0.256170397 |
| *PTRF* | PTRF | Polymerase I and transcript release factor | 0.428245106 |
| *RFTN1* | RFTN1 | Raftlin | 0.49663636 |
| *RPL12* | RL12 | 60S ribosomal protein L12 | 0.350506609 |
| *RPL5* | RL5 | 60S ribosomal protein L5 | 0.034232035 |
| *RPS19* | RS19 | 40S ribosomal protein S19 | 0.322745881 |
| *RPS29* | RS29 | 40S ribosomal protein S29 | 0.166799214 |
| *RPS6* | RS6 | 40S ribosomal protein S6 | 0.239480431 |
| *SDPR* | SDPR | Serum deprivation-response protein | 0.154228706 |
| *SERPINE1* | PAI1 | Plasminogen activator inhibitor 1 | 0.041410659 |
| *SRP14* | SRP14 | Signal recognition particle 14 kDa protein | 0.35196284 |
| *STMN1* | STMN1 | Stathmin | 0.220588803 |
| *STXBP1* | STXB1 | Syntaxin-binding protein 1 | 0.308483423 |
| *THSD4* | THSD4 | Thrombospondin type-1 domain-containing protein 4 | 0.31959279 |
| *TNC* | TENA | Tenascin | 0.227897348 |
| *TUBA1A* | TBA1A | Tubulin alpha-1A chain | 0.118684196 |
| *VIM* | VIME | Vimentin | 0.470064322 |
| *YBX1* | YBOX1 | Nuclease-sensitive element-binding protein 1 | 0.219197834 |
| *YWHAQ* | 1433T | 14-3-3 protein theta | 0.445213335 |
| *ZYX* | ZYX | Zyxin | 0.342835546 |

**GEF-H1-independent FA recruitment (the Ratio of protein abundance 0.5 ~ 2)**

| Gene Name | Entry Name | Common Protein Name | Ratio of Protein Abundance |
|---|---|---|---|
| *A6NL28* | TPM3L | Putative tropomyosin alpha-3 chain-like protein | 0.722166326 |
| *ACTB* | ACTB | Actin, cytoplasmic 1 | 0.576072634 |
| *ACTN1* | ACTN1 | Alpha-actinin-1 | 0.526324022 |
| *ACTN4* | ACTN4 | Alpha-actinin-4 | 0.526324022 |

| | | | |
|---|---|---|---|
| *ADAM9* | ADAM9 | Disintegrin and metalloproteinase domain-containing protein 9 | 1.336957951 |
| *ADAMTS1* | ATS1 | A disintegrin and metalloproteinase with thrombospondin motifs 1 | 0.512533456 |
| *ADD1* | ADDA | Alpha-adducin | 0.587687211 |
| *AGRN* | AGRIN | Agrin | 1.091164547 |
| *AHSG* | FETUA | Alpha-2-HS-glycoprotein | 1.056170458 |
| *ANXA2* | ANXA2 | Annexin A2 | 0.885600181 |
| *AP2M1* | AP2M1 | AP-2 complex subunit mu | 1.370762681 |
| *APOC3* | APOC3 | Apolipoprotein C-III | 0.796138519 |
| *APOE* | APOE | Apolipoprotein E | 0.984948378 |
| *APP* | A4 | Amyloid beta A4 protein | 1.912495812 |
| *ATP2B4* | AT2B4 | Plasma membrane calcium-transporting ATPase 4 | 0.956396362 |
| *BASP1* | BASP1 | Brain acid soluble protein 1 | 0.815250471 |
| *CCDC50* | CCD50 | Coiled-coil domain-containing protein 50 | 0.842445101 |
| *CCDC80* | CCD80 | Coiled-coil domain-containing protein 80 | 1.143852758 |
| *CHCHD3* | CHCH3 | Coiled-coil-helix-coiled-coil-helix domain-containing protein 3, mitochondrial | 1.266128383 |
| *CLTA* | CLCA | Clathrin light chain A | 0.784745884 |
| *CLTB* | CLCB | Clathrin light chain B | 0.951238209 |
| *CNN3* | CNN3 | Calponin-3 | 1.492798216 |
| *COX5A* | COX5A | Cytochrome c oxidase subunit 5A, mitochondrial | 1.408597546 |
| *CSRP1* | CSRP1 | Cysteine and glycine-rich protein 1 | 0.767986275 |
| *CTNNA1* | CTNA1 | Catenin alpha-1 | 0.936604184 |
| *CTNNB1* | CTNB1 | Catenin beta-1 | 1.68855921 |
| *CTTN* | SRC8 | Src substrate cortactin | 0.773244575 |
| *CYR61* | CYR61 | Protein CYR61 | 1.071575188 |
| *DBNL* | DBNL | Drebrin-like protein | 1.59207945 |
| *DKK1* | DKK1 | Dickkopf-related protein 1 | 0.575813819 |
| *EEF1D* | EF1D | Elongation factor 1-delta | 0.930452235 |
| *EEF2* | EF2 | Elongation factor 2 | 0.677891273 |
| *EFEMP2* | FBLN4 | EGF-containing fibulin-like extracellular matrix protein 2 | 0.796431952 |
| *EIF2S1* | IF2A | Eukaryotic translation initiation factor 2 subunit 1 | 1.098853868 |
| *EIF4H* | IF4H | Eukaryotic translation initiation factor 4H | 1.524321019 |
| *EMILIN1* | EMIL1 | EMILIN-1 | 0.61244093 |
| *ENAH* | ENAH | Protein enabled homolog | 1.717313581 |
| *ENSA* | ENSA | Alpha-endosulfine | 1.153570575 |
| *EPB41L2* | E41L2 | Band 4.1-like protein 2 | 0.631966951 |
| *EPHA2* | EPHA2 | Ephrin type-A receptor 2 | 1.025730471 |

| | | | |
|---|---|---|---|
| *EPHB2* | EPHB2 | Ephrin type-B receptor 2 | 1.28029117 |
| *EPN2* | EPN2 | Epsin-2 | 1.000485092 |
| *EZR* | EZRI | Ezrin | 0.54860267 |
| *FAM176B* | F176B | Protein FAM176B | 0.646771609 |
| *FLNA* | FLNA | Filamin-A | 0.65329574 |
| *FLNB* | FLNB | Filamin-B | 0.65329574 |
| *FLNC* | FLNC | Filamin-C | 0.65329574 |
| *FLOT1* | FLOT1 | Flotillin-1 | 0.832435685 |
| *GAPDH* | G3P | Glyceraldehyde-3-phosphate dehydrogenase | 0.52217293 |
| *GLIPR2* | GAPR1 | Golgi-associated plant pathogenesis-related protein 1 | 1.947588144 |
| *GNAI2* | GNAI2 | Guanine nucleotide-binding protein G(i) subunit alpha-2 | 1.372783499 |
| *GNAI3* | GNAI3 | Guanine nucleotide-binding protein G(k) subunit alpha | 1.981700137 |
| *GNG12* | GBG12 | Guanine nucleotide-binding protein G(I)/G(S)/G(O) subunit gamma-12 | 1.062826258 |
| *HBA1* | HBA | Hemoglobin subunit alpha | 0.947245579 |
| *HPCA* | HPCA | Neuron-specific calcium-binding protein hippocalcin | 0.796138519 |
| *HRAS* | RASH | GTPase HRas | 1.504393997 |
| *HSPA9* | GRP75 | Stress-70 protein, mitochondrial | 0.802043021 |
| *IGF2* | IGF2 | Insulin-like growth factor II | 0.719289329 |
| *ITGA2* | ITA2 | Integrin alpha-2 | 0.544310051 |
| *ITGA3* | ITA3 | Integrin alpha-3 | 1.082067691 |
| *ITGA6* | ITA6 | Integrin alpha-6 | 0.943480301 |
| *ITGB1* | ITB1 | Integrin beta-1 | 1.126282401 |
| *ITGB5* | ITB5 | Integrin beta-5 | 0.671486612 |
| *KANK2* | KANK2 | KN motif and ankyrin repeat domain-containing protein 2 | 0.961795091 |
| *LASP1* | LASP1 | LIM and SH3 domain protein 1 | 0.880805856 |
| *LGALS1* | LEG1 | Galectin-1 | 0.703832509 |
| *LIMA1* | LIMA1 | LIM domain and actin-binding protein 1 | 0.547410318 |
| *LIMS2* | LIMS2 | LIM and senescent cell antigen-like-containing domain protein 2 | 1.643661597 |
| *LMNA* | LMNA | Prelamin-A/C | 0.810699289 |
| *LMO7* | LMO7 | LIM domain only protein 7 | 1.776400882 |
| *LOX* | LYOX | Protein-lysine 6-oxidase | 0.848438064 |
| *LTBP2* | LTBP2 | Latent-transforming growth factor beta-binding protein 2 | 1.811514296 |
| *MARCKS* | MARCS | Myristoylated alanine-rich C-kinase substrate | 0.783673946 |
| *MCAM* | MUC18 | Cell surface glycoprotein MUC18 | 1.199014311 |
| *MSN* | MOES | Moesin | 1.042089986 |
| *MT1E* | MT1E | Metallothionein-1E | 0.852735925 |
| *MYH9* | MYH9 | Myosin-9 | 0.999037763 |

| | | | |
|---|---|---|---|
| *MYO1C* | MYO1C | Unconventional myosin-Ic | 1.21605535 |
| *MYOF* | MYOF | Myoferlin | 1.534530491 |
| *NACA* | NACA | Nascent polypeptide-associated complex subunit alpha | 0.66145653 |
| *NDUFA2* | NDUA2 | NADH dehydrogenase [ubiquinone] 1 alpha subcomplex subunit 2 | 1.796433972 |
| *NEXN* | NEXN | Nexilin | 1.825348642 |
| *NPM1* | NPM | Nucleophosmin | 1.113585043 |
| *NRP1* | NRP1 | Neuropilin-1 | 0.773608308 |
| *PDIA3* | PDIA3 | Protein disulfide-isomerase A3 | 1.938120355 |
| *PDLIM2* | PDLI2 | PDZ and LIM domain protein 2 | 1.018690986 |
| *PGAM1* | PGAM1 | Phosphoglycerate mutase 1 | 1.408924164 |
| *PIP4K2A* | PI42A | Phosphatidylinositol 5-phosphate 4-kinase type-2 alpha | 0.690405156 |
| *PKM* | KPYM | Pyruvate kinase isozymes M1/M2 | 0.986144954 |
| *PPFIBP1* | LIPB1 | Liprin-beta-1 | 1.779577733 |
| *PPP1R18* | PPR18 | Phostensin | 1.060973072 |
| *PRKCDBP* | PRDBP | Protein kinase C delta-binding protein | 1.407836628 |
| *PRKCSH* | GLU2B | Glucosidase 2 subunit beta | 1.624910705 |
| *PTK7* | PTK7 | Inactive tyrosine-protein kinase 7 | 0.665117087 |
| *PXN* | PAXI | Paxillin | 1 |
| *RPL22* | RL22 | 60S ribosomal protein L22 | 1.107695318 |
| *RPL35A* | RL35A | 60S ribosomal protein L35a | 1.261150583 |
| *RPLP1* | RLA1 | 60S acidic ribosomal protein P1 | 0.600853012 |
| *RPS21* | RS21 | 40S ribosomal protein S21 | 1.02080179 |
| *RPS25* | RS25 | 40S ribosomal protein S25 | 0.563827082 |
| *RPS3A* | RS3A | 40S ribosomal protein S3a | 1.140860777 |
| *RRBP1* | RRBP1 | Ribosome-binding protein 1 | 0.939000564 |
| *SEPT7* | SEPT7 | Septin-7 | 1.686530689 |
| *SERPINH1* | SERPH | Serpin H1 | 1.026586054 |
| *SLC1A5* | AAAT | Neutral amino acid transporter B(0) | 0.856510264 |
| *SPTAN1* | SPTN1 | Spectrin alpha chain, non-erythrocytic 1 | 0.911650135 |
| *STOM* | STOM | Erythrocyte band 7 integral membrane protein | 0.546356719 |
| *TAGLN2* | TAGL2 | Transgelin-2 | 0.746814773 |
| *TENC1* | TENC1 | Tensin-like C1 domain-containing phosphatase | 1.611921733 |
| *TGFB1* | TGFB1 | Transforming growth factor beta-1 | 0.923798063 |
| *THBS1* | TSP1 | Thrombospondin-1 | 1.112175836 |
| *TLN1* | TLN1 | Talin-1 | 0.71994596 |
| *TNS1* | TENS1 | Tensin-1 | 1.084824308 |
| *TPM3* | TPM3 | Tropomyosin alpha-3 chain | 0.921082916 |

| | | | |
|---|---|---|---|
| *TPM4* | TPM4 | Tropomyosin alpha-4 chain | 1.146858749 |
| *TRIOBP* | TARA | TRIO and F-actin-binding protein | 1.005270576 |
| *TUBB2A* | TBB2A | Tubulin beta-2A chain | 1.45734949 |
| *VASN* | VASN | Vasorin | 0.837225788 |
| *VASP* | VASP | Vasodilator-stimulated phosphoprotein | 1.893070993 |
| *VAT1* | VAT1 | Synaptic vesicle membrane protein VAT-1 homolog | 1.008840908 |
| *VCAN* | CSPG2 | Versican core protein | 0.630305442 |
| *VCL* | VINC | Vinculin | 0.857245078 |
| *VCP* | TERA | Transitional endoplasmic reticulum ATPase | 1.878565552 |
| *WNT5A* | WNT5A | Protein Wnt-5a | 0.543821516 |
| *YWHAZ* | 1433Z | 14-3-3 protein zeta/delta | 0.960747932 |

**GEF-H1-dependent FA recruitment (the Ratio of protein abundance > 2)**

| Gene Name | Entry Name | Common Protein Name | Ratio of Protein Abundance |
|---|---|---|---|
| *AP2A1* | AP2A1 | AP-2 complex subunit alpha-1 | 4.619720557 |
| *CSRP2* | CSRP2 | Cysteine and glycine-rich protein 2 | 2.204501867 |
| *DBN1* | DREB | Drebrin | 3.970404849 |
| *EEF1A1* | EF1A1 | Elongation factor 1-alpha 1 | 3.459791412 |
| *FBLN1* | FBLN1 | Fibulin-1 | 7.432235289 |
| *FBLN2* | FBLN2 | Fibulin-2 | 2.053948691 |
| *HAPLN3* | HPLN3 | Hyaluronan and proteoglycan link protein 3 | 2.664250221 |
| *HSPA8* | HSP7C | Heat shock cognate 71 kDa protein | 7.946151851 |
| *INA* | AINX | Alpha-internexin | 2.982156035 |
| *LIMD1* | LIMD1 | LIM domain-containing protein 1 | 3.252673709 |
| *LIN7C* | LIN7C | Protein lin-7 homolog C | 2.685179485 |
| *LRP1* | LRP1 | Prolow-density lipoprotein receptor-related protein 1 | 2.913148472 |
| *MYL6* | MYL6 | Myosin light polypeptide 6 | 4.124270925 |
| *PLEC* | PLEC | Plectin | 2.693428906 |
| *RPL11* | RL11 | 60S ribosomal protein L11 | 2.667937446 |
| *RPL13* | RL13 | 60S ribosomal protein L13 | 2.682377789 |
| *RPL7A* | RL7A | 60S ribosomal protein L7a | 5.800140454 |
| *RPS18* | RS18 | 40S ribosomal protein S18 | 2.132765586 |
| *RPS2* | RS2 | 40S ribosomal protein S2 | 2.121090266 |
| *SCAMP3* | SCAM3 | Secretory carrier-associated membrane protein 3 | 2.101951701 |
| *SEPT2* | SEPT2 | Septin-2 | 2.394787005 |
| *SEPT8* | SEPT8 | Septin-8 | 2.484090993 |

| | | | |
|---|---|---|---|
| *SEPT9* | SEPT9 | Septin-9 | 3.162943249 |
| *SH3BGRL3* | SH3L3 | SH3 domain-binding glutamic acid-rich-like protein 3 | 2.165327453 |
| *SPTBN1* | SPTB2 | Spectrin beta chain, non-erythrocytic 1 | 4.251727902 |
| *SRP9* | SRP09 | Signal recognition particle 9 kDa protein | 2.111988128 |
| *TJP2* | ZO2 | Tight junction protein ZO-2 | 3.405246567 |
| *TPI1* | TPIS | Triosephosphate isomerase | 2.998747322 |
| *TPM2* | TPM2 | Tropomyosin beta chain | 8.706196131 |
| *UTRN* | UTRO | Utrophin | 4.040178085 |
| *VTN* | VTNC | Vitronectin | 2.962236304 |
| *ZC3H15* | ZC3HF | Zinc finger CCCH domain-containing protein 15 | 2.752724594 |

**Only present in non-silencing FAs**

| Gene Name | Entry Name | Common Protein Name |
|---|---|---|
| *ADAM5P* | ADAM5 | Putative disintegrin and metalloproteinase domain-containing protein 5 |
| *ADRM1* | ADRM1 | Proteasomal ubiquitin receptor ADRM1 |
| *APOH* | APOH | Beta-2-glycoprotein 1 |
| *ARHGEF2* | ARHG2 | Rho guanine nucleotide exchange factor 2 |
| *ARPC5* | ARPC5 | Actin-related protein 2/3 complex subunit 5 |
| *ATP5D* | ATPD | ATP synthase subunit delta, mitochondrial |
| *ATP5I* | ATP5I | ATP synthase subunit e, mitochondrial |
| *ATP5O* | ATPO | ATP synthase subunit O, mitochondrial |
| *BCAM* | BCAM | Basal cell adhesion molecule |
| *C16orf55* | CP055 | Uncharacterized protein C16orf55 |
| *CD151* | CD151 | CD151 antigen |
| *CD55* | DAF | Complement decay-accelerating factor |
| *CD59* | CD59 | CD59 glycoprotein |
| *CD99* | CD99 | CD99 antigen |
| *CDC42EP1* | BORG5 | Cdc42 effector protein 1 |
| *CDC42EP3* | BORG2 | Cdc42 effector protein 3 |
| *CKAP4* | CKAP4 | Cytoskeleton-associated protein 4 |
| *CLEC3B* | TETN | Tetranectin |
| *CLTC* | CLH1 | Clathrin heavy chain 1 |
| *CNP* | CN37 | 2',3'-cyclic-nucleotide 3'-phosphodiesterase |
| *CTGF* | CTGF | Connective tissue growth factor |
| *CTNND1* | CTND1 | Catenin delta-1 |
| *DDX3X* | DDX3X | ATP-dependent RNA helicase DDX3X |

| | | |
|---|---|---|
| *EGFR* | EGFR | Epidermal growth factor receptor |
| *EHD1* | EHD1 | EH domain-containing protein 1 |
| *EHD4* | EHD4 | EH domain-containing protein 4 |
| *EPS15L1* | EP15R | Epidermal growth factor receptor substrate 15-like 1 |
| *ERBB2IP* | LAP2 | Protein LAP2 |
| *FBLIM1* | FBLI1 | Filamin-binding LIM protein 1 |
| *FERMT2* | FERM2 | Fermitin family homolog 2 |
| *GAS6* | GAS6 | Growth arrest-specific protein 6 |
| *GNAS* | GNAS1 | Guanine nucleotide-binding protein G(s) subunit alpha isoforms XLas |
| *HAPLN1* | HPLN1 | Hyaluronan and proteoglycan link protein 1 |
| *HNRNPA1* | ROA1 | Heterogeneous nuclear ribonucleoprotein A1 |
| *HNRNPA2B1* | ROA2 | Heterogeneous nuclear ribonucleoproteins A2/B1 |
| *HNRNPD* | HNRPD | Heterogeneous nuclear ribonucleoprotein D0 |
| *ITGA7* | ITA7 | Integrin alpha-7 |
| *KIRREL* | KIRR1 | Kin of IRRE-like protein 1 |
| *KPNB1* | IMB1 | Importin subunit beta-1 |
| *LPP* | LPP | Lipoma-preferred partner |
| *LSM12* | LSM12 | Protein LSM12 homolog |
| *MACF1* | MACF1 | Microtubule-actin cross-linking factor 1, isoforms 1/2/3/5 |
| *MRC2* | MRC2 | C-type mannose receptor 2 |
| *MT2A* | MT2 | Metallothionein-2 |
| *MYH10* | MYH10 | Myosin-10 |
| *MYL9* | MYL9 | Myosin regulatory light polypeptide 9 |
| *NUMB* | NUMB | Protein numb homolog |
| *PDLIM4* | PDLI4 | PDZ and LIM domain protein 4 |
| *PFN2* | PROF2 | Profilin-2 |
| *PHLDB1* | PHLB1 | Pleckstrin homology-like domain family B member 1 |
| *PLP2* | PLP2 | Proteolipid protein 2 |
| *PLSCR3* | PLS3 | Phospholipid scramblase 3 |
| *PLXNB2* | PLXB2 | Plexin-B2 |
| *PPIB* | PPIB | Peptidyl-prolyl cis-trans isomerase B |
| *PPP1R12A* | MYPT1 | Protein phosphatase 1 regulatory subunit 12A |
| *PRDX1* | PRDX1 | Peroxiredoxin-1 |
| *PRSS12* | NETR | Neurotrypsin |
| *PVR* | PVR | Poliovirus receptor |
| *RAB13* | RAB13 | Ras-related protein Rab-13 |
| *RAB21* | RAB21 | Ras-related protein Rab-21 |

| | | |
|---|---|---|
| *RAC1* | RAC1 | Ras-related C3 botulinum toxin substrate 1 |
| *RAI14* | RAI14 | Ankycorbin |
| *RPL23A* | RL23A | 60S ribosomal protein L23a |
| *RPL29* | RL29 | 60S ribosomal protein L29 |
| *RPL30* | RL30 | 60S ribosomal protein L30 |
| *RPL6* | RL6 | 60S ribosomal protein L6 |
| *RPS28* | RS28 | 40S ribosomal protein S28 |
| *S100A11* | S10AB | Protein S100-A11 |
| *SERBP1* | PAIRB | Plasminogen activator inhibitor 1 RNA-binding protein |
| *SLC39A14* | S39AE | Zinc transporter ZIP14 |
| *SLC7A5* | LAT1 | Large neutral amino acids transporter small subunit 1 |
| *SNAP29* | SNP29 | Synaptosomal-associated protein 29 |
| *SNTB2* | SNTB2 | Beta-2-syntrophin |
| *STEAP3* | STEA3 | Metalloreductase STEAP3 |
| *STX7* | STX7 | Syntaxin-7 |
| *TFRC* | TFR1 | Transferrin receptor protein 1 |
| *TGFB1I1* | TGFI1 | Transforming growth factor beta-1-induced transcript 1 protein |
| *TGFB2* | TGFB2 | Transforming growth factor beta-2 |
| *TLN2* | TLN2 | Talin-2 |
| *TMOD3* | TMOD3 | Tropomodulin-3 |
| *TRIP6* | TRIP6 | Thyroid receptor-interacting protein 6 |
| *TUFM* | EFTU | Elongation factor Tu, mitochondrial |
| *UACA* | UACA | Uveal autoantigen with coiled-coil domains and ankyrin repeats |
| *UQCRC1* | QCR1 | Cytochrome b-c1 complex subunit 1, mitochondrial |
| *VAMP5* | VAMP5 | Vesicle-associated membrane protein 5 |

**Table S4: The list of shRNA, siRNA, primers and antibodies used in the experiments.**

**shRNA and siRNA**

| Name | Vector | Target sequence |
|---|---|---|
| GEF-H1 shRNA | pLenti6/BLOCK-iT0222-DEST (Invitrogen) | GTGACTATCCACAACCGCTGT |
| NMIIB shRNA | pLKO.1 (the National RNAi Core Facility Plateform, Taiwan) | GCCAAGCTCAAGAACAAGCAT |
| GEF-H1 siRNA (Dharmacon_Thermo Science) | | CAACGGAACUGGCAUUACU |

**Primers**

| Name | Sequence |
|---|---|
| the forward PCR primer of GEF-H1 for GFP-C1-GEF-H1 | 5'- AATTCAAGCTTTAATGTCTCGGATCGAATCCCTCACGCGGGCG-3' |
| the reverse PCR primer of GEF-H1 for GFP-C1-GEF-H1 | 5'- AATTCGGTACCTTAGCTCTCGGAGGCTACAGCCTCCCCGTC-3' |
| the site-directed mutagenesis primer for pGFP-C1-GEF-H1 (shRNA resistance) | 5'-CTGCAATGTGACTATCCATAATCGCTGTAAAGACA-3' |
| the forward PCR primer of GEF-H1$_{1\text{-}235}$ for GFP-C1-GEF-H1_DH(m) | 5'- AATTCAAGCTTTAATGTCTCGGATCGAATCCCTCACGCGGGCG-3' |
| the reverse PCR primer of GEF-H1$_{1\text{-}235}$ for GFP-C1-GEF-H1_DH(m) | 5'-AATTCCCATGGGAGGGTATTTATCAGCTGGAGAAAGGGGCC-3' |
| the forward PCR primer of GEF-H1$_{429\text{-}985}$ for GFP-C1-GEF-H1_DH(m) | 5'- AATTCCCATGGCTTCATCACCTCCTTTTTATGCTGCTGCAG-3' |
| the reverse PCR primer of GEF-H1$_{429\text{-}985}$ for GFP-C1-GEF-H1_DH(m) | 5'- AATTCGGTACCTTAGCTCTCGGAGGCTACAGCCTCCCCGTC-3' |
| the site-directed mutagenesis primer for GFP-C1-GEF-H1_DH (m) (shRNA resistance) | 5'-GTGACTATCCATAATCGCTGT-3' |
| the forward PCR primer for pLKO-AS3W-GEF-H1 and pLKO-AS3W-GEF-H1_DH (m) | 5'- AATTCGCTAGCATGTCTCGGATCGAATCCCTCACGCGGGCG-3' |
| the reverse PCR primer for pLKO-AS3W-GEF-H1 and pLKO-AS3W-GEF-H1_DH (m) | 5'- AATTCGAATTCCCTTAGCTCTCGGAGGCTACAGCCTCCCCGTC-3' |

**Antibodies**

| Name | Host | Catalog number | Dilutions |
|---|---|---|---|
| paxillin | mouse | BD 610052 | dilution for western: 1/5000<br>dilution for immunofluorescence: 1/1000 |

| | | | |
|---|---|---|---|
| phospho-MLC (Thr18/Ser19) | rabbit | Cell signaling #3674 | dilution for western: 1/1000 |
| MLC | rabbit | Cell Signaling #3672 | dilution for western: 1/1000 |
| GAPDH | mouse | GeneTex GTX627408 | dilution for western: 1/1000 |
| GAPDH | rabbit | GeneTex GTX100118 | dilution for IP: 1/100 |
| GEF-H1 | rabbit | GeneTex GTX125893 | dilution for western: 1/1000<br>dilution for immunofluorescence: 1/50<br>dilution for IP: 1/100 |
| integrin β1 | mouse | Millipore MAB2259Z | dilution for western: 1/1000 |
| NMIIA | rabbit | Cell signaling #3403 | dilution for western: 1/1000 |
| NMIIB | rabbit | Cell signaling #3404 | dilution for western: 1/1000<br>dilution for immunofluorescence: 1/100 |
| GFP | rabbit | GeneTex GTX113617 | dilution for western: 1/2500 |
| Alexa Fuor 568-anti-mouse IgG | | Invitrogen A11031 | dilution for immunofluorescence: 1/300 |
| Alexa Fuor 488-anti-rabbit IgG | | Invitrogen A11034 | dilution for immunofluorescence: 1/300 |
| Alexa Fuor 647-anti-mouse IgG | | Jackson ImmunoResearch 715-606-151/J1 | dilution for immunofluorescence: 1/300 |
| Alexa Fluor 488 phalloidin | | Invitrogen A12379 | dilution for immunofluorescence: 1/400 |
| HRP-AffiniPure goat anti-mouse IgG | | Jackson ImmunoResearch 115-035-174 | dilution for immunofluorescence: 1/5000 |
| HRP-AffiniPure goat anti-rabbit IgG | | Jackson ImmunoResearch 211-032-171 | dilution for immunofluorescence: 1/5000 |
| GFP-Trap beads | | ChromoTek gta-100 | |
